# Extracellular Matrix Proteomics Reveals Interplay of Aggrecan and Aggrecanases in Vascular Remodeling of Stented Coronary Arteries

**DOI:** 10.1161/CIRCULATIONAHA.116.023381

**Published:** 2018-01-08

**Authors:** Gonca Suna, Wojciech Wojakowski, Marc Lynch, Javier Barallobre-Barreiro, Xiaoke Yin, Ursula Mayr, Ferheen Baig, Ruifang Lu, Marika Fava, Robert Hayward, Chris Molenaar, Stephen J. White, Tomasz Roleder, Krzysztof P. Milewski, Pawel Gasior, Piotr P. Buszman, Pawel Buszman, Marjan Jahangiri, Catherine M. Shanahan, Jonathan Hill, Manuel Mayr

**Affiliations:** 1 King’s British Heart Foundation Centre, King’s College London, United Kingdom (G.S., M.L., J.B.-B., X.Y., U.M., F.B., R.L., M.F., R.H., C.M., C.M.S., M.M.); 2 3rd Division of Cardiology, Medical University of Silesia, Katowice, Poland (W.W., T.R., P.G.); 3 Healthcare Science Research Centre, Manchester Metropolitan University, United Kingdom (S.J.W.); 4 Centre for Cardiovascular Research and Development, American Heart of Poland, Katowice (K.P.M., P.P.B., P.B.); 5 St George’s Vascular Institute, St George’s Healthcare NHS Trust, London, United Kingdom (M.J.); 6 King’s College Hospital and King’s Health Partners Academic Health Sciences, London, United Kingdom (J.H.).

**Keywords:** coronary artery, disease, extracellular matrix, mass spectrometry, neointima, stents

## Abstract

**Background:**

Extracellular matrix (ECM) remodeling contributes to in-stent restenosis and thrombosis. Despite its important clinical implications, little is known about ECM changes post–stent implantation.

**Methods:**

Bare-metal and drug-eluting stents were implanted in pig coronary arteries with an overstretch under optical coherence tomography guidance. Stented segments were harvested 1, 3, 7, 14, and 28 days post-stenting for proteomics analysis of the media and neointima.

**Results:**

A total of 151 ECM and ECM-associated proteins were identified by mass spectrometry. After stent implantation, proteins involved in regulating calcification were upregulated in the neointima of drug-eluting stents. The earliest changes in the media were proteins involved in inflammation and thrombosis, followed by changes in regulatory ECM proteins. By day 28, basement membrane proteins were reduced in drug-eluting stents in comparison with bare-metal stents. In contrast, the large aggregating proteoglycan aggrecan was increased. Aggrecanases of the ADAMTS (a disintegrin and metalloproteinase with thrombospondin motifs) family contribute to the catabolism of vascular proteoglycans. An increase in ADAMTS-specific aggrecan fragments was accompanied by a notable shift from *ADAMTS1* and *ADAMTS5* to *ADAMTS4* gene expression after stent implantation. Immunostaining in human stented coronary arteries confirmed the presence of aggrecan and aggrecan fragments, in particular, at the contacts of the stent struts with the artery. Further investigation of aggrecan presence in the human vasculature revealed that aggrecan and aggrecan cleavage were more abundant in human arteries than in human veins. In addition, aggrecan synthesis was induced on grafting a vein into the arterial circulation, suggesting an important role for aggrecan in vascular plasticity. Finally, lack of ADAMTS-5 activity in mice resulted in an accumulation of aggrecan and a dilation of the thoracic aorta, confirming that aggrecanase activity regulates aggrecan abundance in the arterial wall and contributes to vascular remodeling.

**Conclusions:**

Significant differences were identified by proteomics in the ECM of coronary arteries after bare-metal and drug-eluting stent implantation, most notably an upregulation of aggrecan, a major ECM component of cartilaginous tissues that confers resistance to compression. The accumulation of aggrecan coincided with a shift in *ADAMTS* gene expression. This study provides the first evidence implicating aggrecan and aggrecanases in the vascular injury response after stenting.

## Abstract 2

Supplemental Digital Content is available in the text.

**Clinical Perspective**

**What Is New?**

- Despite continuous development in stent technology, in-stent restenosis and neoatherosclerosis remain a considerable cause of stent failure.
- A proteomics approach to identify extracellular matrix protein changes in response to bare-metal stent and drug-eluting stent insertion revealed differential expression of aggrecan, a proteoglycan that is usually associated with articular cartilage.
- Aggrecanase activity is part of the vascular injury response post-stenting.

**What Are the Clinical Implications?**

- Aggrecan cleavage is the hallmark of cartilage degeneration in a number of degenerative diseases including rheumatoid arthritis and osteoarthritis.
- Aggrecanase activity might offer new drug targets to alter extracellular matrix remodeling in the vasculature.

Percutaneous coronary intervention with stent implantation has become the most widely used treatment for coronary artery disease. Use of stents reduced the impact of acute elastic recoil and occlusive dissection on restenosis observed with balloon angioplasty.^1,2^ However, stent implantation induced an excessive healing process, which, in the case of bare-metal stents (BMS), led to neointimal hyperplasia and in-stent restenosis. The introduction of drug-eluting stents (DES) reduced the burden of in-stent restenosis and repeat revascularization.^3,4^ However, DES implantation delays vessel reendothelialization because of the nonselective nature of these drugs, resulting in an increased risk of late and very late stent thrombosis requiring prolonged dual antiplatelet therapy.^5^ Late stent thrombosis has also been associated with the inflammation and hypersensitivity reactions to the nonbiocompatible polymer coatings on DES.^6^ Moreover, in the long term, neoatherosclerosis is likely to occur earlier in DES than in BMS.^7^ Second-generation DES with durable biocompatible polymers are the current standard of care because of their excellent efficacy and safety. A further development in stent technology uses biodegradable polymers and fully biodegradable nonmetallic scaffolds, as well.^8,9^

However, despite continuous development in technology, in-stent restenosis remains a considerable cause of stent failure.^10^ Neointimal hyperplasia involves the interaction between inflammatory cells, platelets, smooth muscle cells (SMCs), and endothelial cells (ECs) leading to progressive obliteration of the vascular lumen.^11^ Platelet activation and inflammation as an early response to stent deployment result in increased SMC migration, proliferation, and extracellular matrix (ECM) production. Yet, little is known about changes in ECM composition on vascular stent injury, even though >50% of the neointimal hyperplasia consists of ECM proteins.^12^ Our current knowledge of ECM remodeling after stent injury and during neointima formation is mainly based on histopathologic analysis investigating the role of only few selected ECM proteins.^12,13^ In comparison, proteomics is a powerful underpinning technology to profile not just individual ECM proteins by antibody staining, but to characterize the different stages of ECM remodeling by liquid chromatography tandem mass spectrometry (LC-MS/MS).

Therefore, the aim of the present study was to use proteomics to characterize ECM remodeling in porcine coronary arteries stented with BMS and DES. Pigs are well suited for these studies, because the stages of the healing process closely resemble the human disease, but the time course of neointimal hyperplasia formation is notably shorter.^14,15^

## Methods

An expanded Methods is available in the online-only Data Supplement. The data, analytic methods, and study materials will not be made available to other researchers for purposes of reproducing the results or replicating the procedure because of the limited amount of tissues. The proteomics data, however, are deposited in PRIDE^16^ with the data set identifiers PXD005726 and 10.6019/PXD005726.

### Porcine Model of Stent Injury

All porcine animal procedures were approved by the local ethical committee for animal experiments, Institute of Pharmacology Polish Academy of Science, Cracow, Poland. Twelve healthy male and female domestic pigs (3–4 months old, 28–48 kg) underwent percutaneous coronary intervention through transfemoral access. To prevent the risk of in-stent thrombosis, pigs received a loading dose of aspirin and clopidogrel orally 24 hours before intervention and remained on this dual antiplatelet therapy until termination. The coronary arteries of each animal (left anterior descending, left circumflex, and right coronary arteries) were either stented with a BMS (MULTI-LINK cobalt-chromium stent, Abbott Vascular) or a DES based on the same cobalt-chromium platform (XIENCE PRO everolimus-eluting stent with durable fluoropolymer, Abbott Vascular) or had balloon angioplasty (BA) alone. Full strut expansion was achieved for each deployed stent. Quantitative coronary angiography and optical coherence tomography (OCT) were performed 1, 3, 7, 14, or 28 days post–stent implantation, followed by harvesting of the coronary arteries. The neointima lesions that had developed in stented arteries at day 28 were dissected from the media and analyzed separately. In total, 31 samples were analyzed by LC-MS/MS for the media (n=3 BMS and n=3 DES at each time point 1, 3, 7, and 28 days; n=4 BA early [day 1 to day 3] and n=3 BA late [day 14 to day 28]). For the neointima, a total of 14 samples were analyzed (n=7 BMS, n=7 DES at 28 days). Six coronary arteries of 4.5-month-old healthy pigs were harvested as unstented controls.

### ECM Extraction

The ECM proteins for the media were extracted in a 3-step manner with a method previously developed in our laboratory for the enrichment of ECM proteins.^17^ In brief, newly synthesized, loosely bound ECM proteins were extracted in 0.5 mol/L NaCl (1.5 hours), followed by decellularization of the tissue in 0.08% sodium dodecyl sulfate (1.5 hours) to remove intracellular material. Eventually the tissue pieces were incubated in 4 mol/L guanidine HCl buffer (48 hours) by vigorous shaking at room temperature to extract the strongly bound ECM components. The buffers contained protease inhibitors and EDTA to prevent ECM protein degradation by proteases. Proteins of the thinner neointimal tissues were extracted only in guanidine HCl buffer for 48 hours.

### Proteomics Analysis in Porcine Tissue

After deglycosylation, proteins were subjected to in-solution digestion and analyzed by LC-MS/MS. Fifteen micrograms of deglycosylated proteins of the guanidine HCl extracts were denatured with 6 mol/L urea and 2 mol/L thiourea, reduced with 10 mmol/L dichlorodiphenyltrichloroethane, and alkylated with 50 mmol/L iodoacetamide, followed by acetone precipitation of the proteins and tryptic digestion overnight. Subsequently, peptides were cleaned up using C18 spin plates and separated on a nanoflow high-performance LC column (Acclaim, ThermoFisher) using an UltiMate 3000 RSLCnano LC system (ThermoFisher) interfaced to a Q Exactive Plus Orbitrap mass spectrometer (ThermoFisher). MS/MS analysis was performed on the 15 most abundant ions in each full MS scan with dynamic exclusion enabled.^18^ Raw files were searched using Proteome Discoverer 1.4 against a custom-made database, containing porcine ECM proteins with a human proteome background (UniProtKB/Swiss-Prot Release 2014_06, 20220 protein entries) using Mascot. All data were exported to mzIdentML format using Scaffold and deposited to the ProteomeXchange Consortium via the PRIDE partner repository.

### Immunohistochemistry in Human Specimen

Human samples were collected under the Bristol Coronary Biobank ethical approval 08/H0107/48. The artery including stent was fixed in 10% formalin, then the stent was removed, and the tissues were paraffin embedded and sectioned at 3-µm thickness by using a microtome. For immunostaining, deparaffinized and rehydrated samples were incubated in 0.3% H_2_O_2_ for 10 minutes to block endogenous peroxidases, then incubated in a boiling water bath in preheated 10 mmol/L sodium citrate buffer (pH 6.0) for antigen retrieval. Sections were blocked with either 20% goat (for rabbit primary) or rabbit serum (for goat primary) in phosphate-buffered saline for 1 hour and subsequently incubated with primary antibody or matched rabbit IgG control (5 µg/mL) in 1% goat or rabbit serum and incubated at 4°C overnight. Primary antibodies were used to aggrecan (1:400; Abcam, ab36861), the NITEGE neoepitope of aggrecan (1:400; Thermo, AF-PA11746), hyaluronan and proteoglycan link protein 1 (HPLN1) (1:200; Abcam, ab103455), the DPEAAE neoepitope of versican (1:400; Abcam, ab19345), matrix Gla protein (MGP) (1:100; Abcam, ab86233), and secreted phosphoprotein 24 (SPP24) (1:50; sc-169408, Santa Cruz). Biotinylated goat anti-rabbit (1:250; Sigma, B7389) or rabbit anti-goat (1:250; Dako, E0466) secondary antibodies were applied for 1 hour, followed by wash steps and Extravidin-HRP (1:250; Sigma, E2886) incubation. 3,3′-Diaminobenzidine tetrahydrochloride solution (Vector) for 10 minutes was used for detection. Sections were washed in dH_2_O, counterstained for 15 seconds in modified Harris Hematoxylin, dehydrated, and mounted in DPX. Histological slides were digitally scanned using a digital scanning system (LEICA SCN400F) to provide a high-resolution digital image.

### Statistical Analysis

Data are shown as average±standard error of the mean. MS data were quantified using ion intensities (total ion current). IBM SPSS statistics software (version 22) and Microsoft Excel (version 15.20) were used for statistical calculations, GraphPad Prism (version 6.0e) and Microsoft Excel (version 15.20) for data illustration. For OCT analysis, 2-way analysis of variance was applied to assess neointimal volume, minimal lumen area, and strut coverage between BMS and DES at different time points. One-way analysis of variance was applied for changes in protein abundance and gene expression in BMS and DES at different time points. Unpaired Student *t* test with unequal variance was applied for proteomics differences between neointimal BMS and DES, BA early and late, for differences between BMS and DES at each time point (visualized as a volcano plot for changes at day 28), and the comparison between BMS or DES day 28 versus BA late, as well. Unpaired, 2-tailed Student *t* test with unequal variance was also used for the proteomics data in mice, and comparisons on murine aortic diameter, blood pressure, and cardiac output, as well. MultiExperiment Viewer software (MeV, version v4.9) was applied using Pearson correlation for protein clustering. For gene expression analysis of porcine tissue, *t* tests were applied for differences between BMS and DES and regression analysis for changes at different time points (*P* value for trend). Student *t* test with unequal variance was used for densitometry quantification of immunoblots. Correction for multiple comparisons was performed using the Benjamini-Hochberg procedure,^19^ controlling the false discovery rate. Uncorrected *P* values and false discovery rate are presented in the tables. *P* values <0.05 were considered significant. A false discovery rate threshold of 10% was applied.

## Results

### Proteomics Analysis of a Porcine Model of Stent Injury

**Figure 1.**
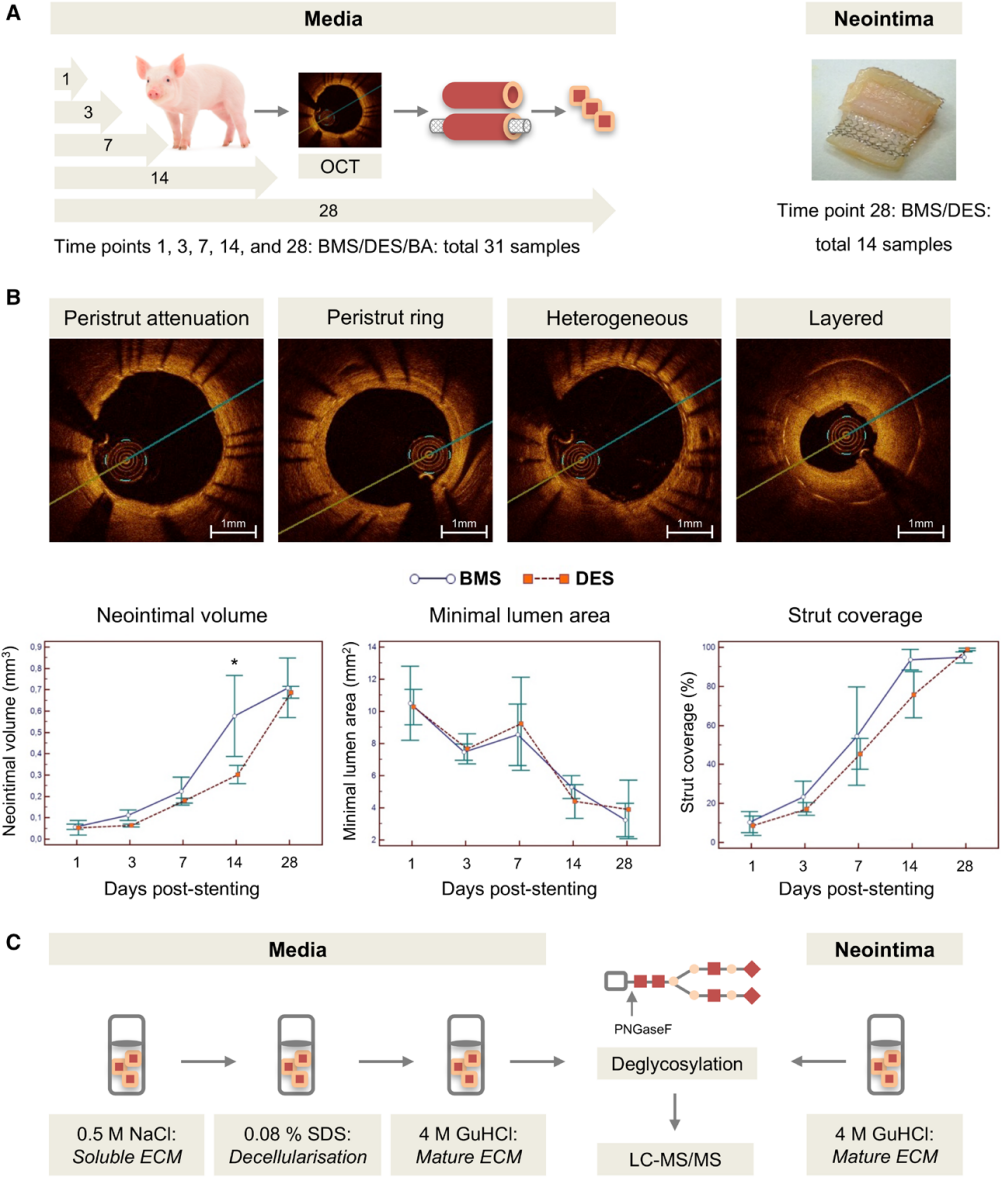
**Porcine model of stent injury.** **A**, Pigs underwent PCI with BMS/DES/BA treatment. Coronary arteries were retrieved for proteomics at 1, 3, 7, 14, and 28 days after stent deployment. The evolved neointimal lesions at day 28 were analyzed separately. In total, 45 samples were analyzed by LC-MS/MS. **B**, Quantitative analysis of the neointima was performed by OCT. The neointimal structure was classified into homogeneous with peristrut attenuation/ring, heterogeneous, or layered. At 14 days post–stent implantation, neointimal volume was higher in BMS than in DES (*P=0.025) with no significant difference at day 28. *P*=0.89 (2-way ANOVA). **C**, ECM proteins were obtained using our previously published extraction procedure, followed by LC-MS/MS analysis of the deglycosylated tryptic peptides. BA indicates balloon angioplasty; BMS, bare-metal stent; DES, drug-eluting stent; ECM, extracellular matrix; GuHCl, guanidine HCl buffer; LC-MS/MS, liquid chromatography tandem mass spectrometry; OCT, optical coherence tomography; PCI, percutaneous coronary intervention; SDS, sodium dodecyl sulfate.

BMS or DES were implanted in porcine coronary arteries. Coronary arteries subjected to BA alone without stent deployment served as controls (Figure 1A). OCT was performed at 5 time points 1, 3, 7, 14, and 28 days post–stent implantation (Figure 1B). The OCT analysis revealed that, at 14 days post–stent implantation, neointimal volume was higher in BMS than in DES (*P*=0.025) with no significant difference at day 28. The minimal lumen area and the stent strut coverage were not different for BMS and DES during the 28-day follow-up. Time points without significant differences were chosen for proteomic analysis to identify differences in the ECM composition. In total, 45 samples were analyzed by proteomics (Figure I in the online-only Data Supplement). Characteristics of all samples are summarized in Table I in the online-only Data Supplement.

A 3-step extraction method as previously described was used to sequentially extract the ECM proteins of the vascular media.^16^ Twenty-eight days post–stent implantation, a neointima had evolved on top of the stent struts regardless of the stent type. The neointima was separated from the media before proteomics analyses. Following deglycosylation and tryptic digestion, proteomics analysis of the neointima and the media was performed by LC-MS/MS (Figure 1C). Duplicate measurements performed on the neointima samples showed good reproducibility for spectral counts and sequence coverage, and number of identified peptides, as well (Figure II in the online-only Data Supplement).

To improve the identification of ECM proteins on MS, a custom-made pig ECM protein database was generated for a database search containing a comprehensive porcine sequence list of previously published ECM proteins (Figure III in the online-only Data Supplement). A total of 151 unique ECM proteins were identified overall in the neointima and the media with a minimum of 2 high-confidence peptides (Figure IV in the online-only Data Supplement, Tables II through IV in the online-only Data Supplement). Proteins only identified in the media (n=11) or neointima (n=26) are marked with an asterisk.

### Comparison of the Neointima of DES and BMS

**Figure 2.**
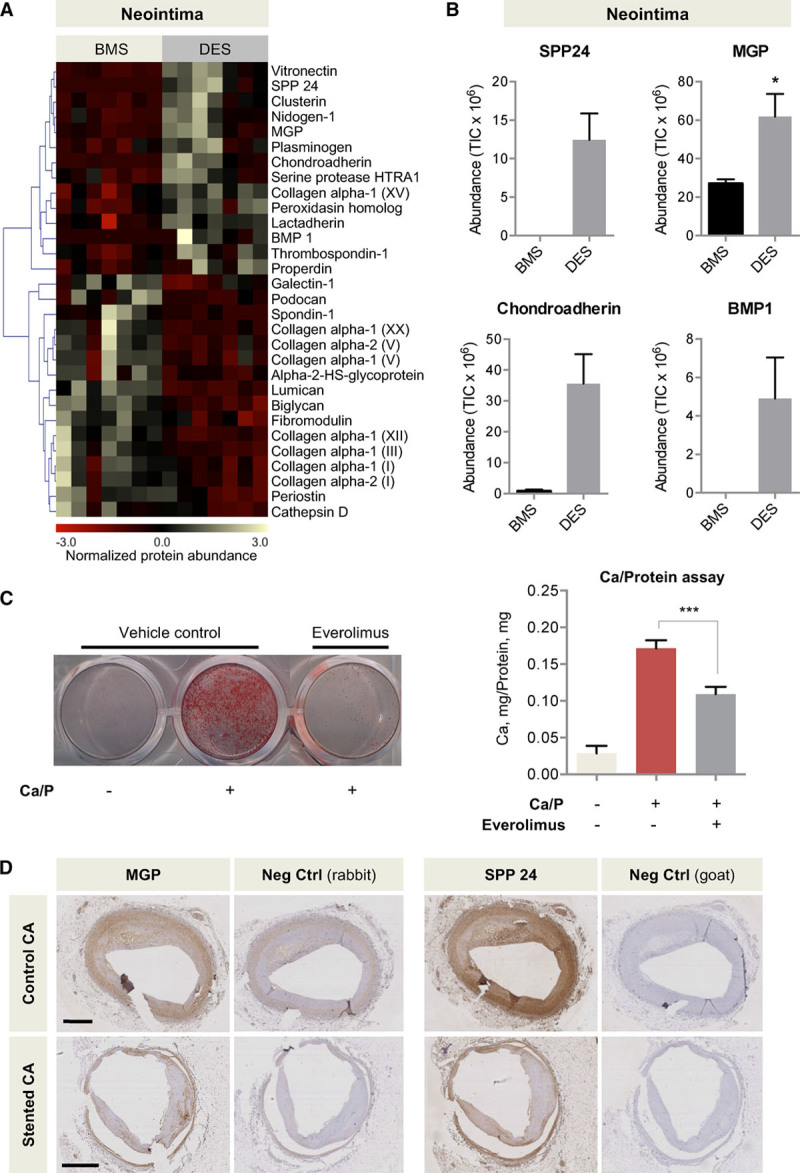
**ECM remodeling in the neointima.** **A**, Heat map of proteins with differential abundance. n=7 per group (*t* test with unequal variance). **B**, Proteins involved in the regulation of calcification (SPP24, MGP, BMP1) and chondroadherin were predominantly found in the DES group. *P<0.05. BMP 1 and SPP24 were undetectable in the control group, and chondroadherin was undetectable in the majority of the samples in the control group, thus a *t* test was not performed. **C**, Everolimus treatment reduced SMC calcification as revealed by Alizarin Red staining and *o*-cresolphthalein complexone assay. n=triplicates of 4 independent experiments. ***P<0.001 (*t* test with unequal variance). **D**, MGP and SPP24 in human stented and control coronary arteries. Scale bars=1 mm. BMP1 indicates bone morphogenetic protein 1; BMS, bare-metal stent; CA, coronary artery; Ctrl, control; DES, drug-eluting stent; ECM, extracellular matrix; MGP, matrix Gla protein; SMC, smooth muscle cell; SPP24, secreted phosphoprotein 24; and TIC, total ion current.

In comparison with BMS, the neointima of arteries with DES implantation contained fewer structural constituents of the ECM like collagen type I, III, V, and regulatory ECM proteins, such as biglycan, lumican, fibromodulin, or periostin, as well (Figure 2A); in contrast, proteins involved in the regulation of calcification such as MGP, SPP24, and bone morphogenetic protein 1 were increased (Figure 2B). It is interesting to note that proteomics also uncovered the presence of chondroadherin in coronary arteries with DES (Figure 2B). This small leucine-rich proteoglycan (SLRP) has not been previously detected in the vasculature at the protein level. Next, we tested whether everolimus treatment had an effect on calcification of human aortic SMCs in culture using the *o*-cresolphthalein assay (Figure 2C). Everolimus was coated on the DES used in porcine coronary arteries. Indeed, an inhibition of calcification was observed on everolimus treatment. Immunohistochemistry localized both MGP and SPP24 in human coronary arteries with atherosclerotic plaques with and without stent implantation (Figure 2D).

### ECM Changes in the Media Post-Stenting

**Figure 3.**
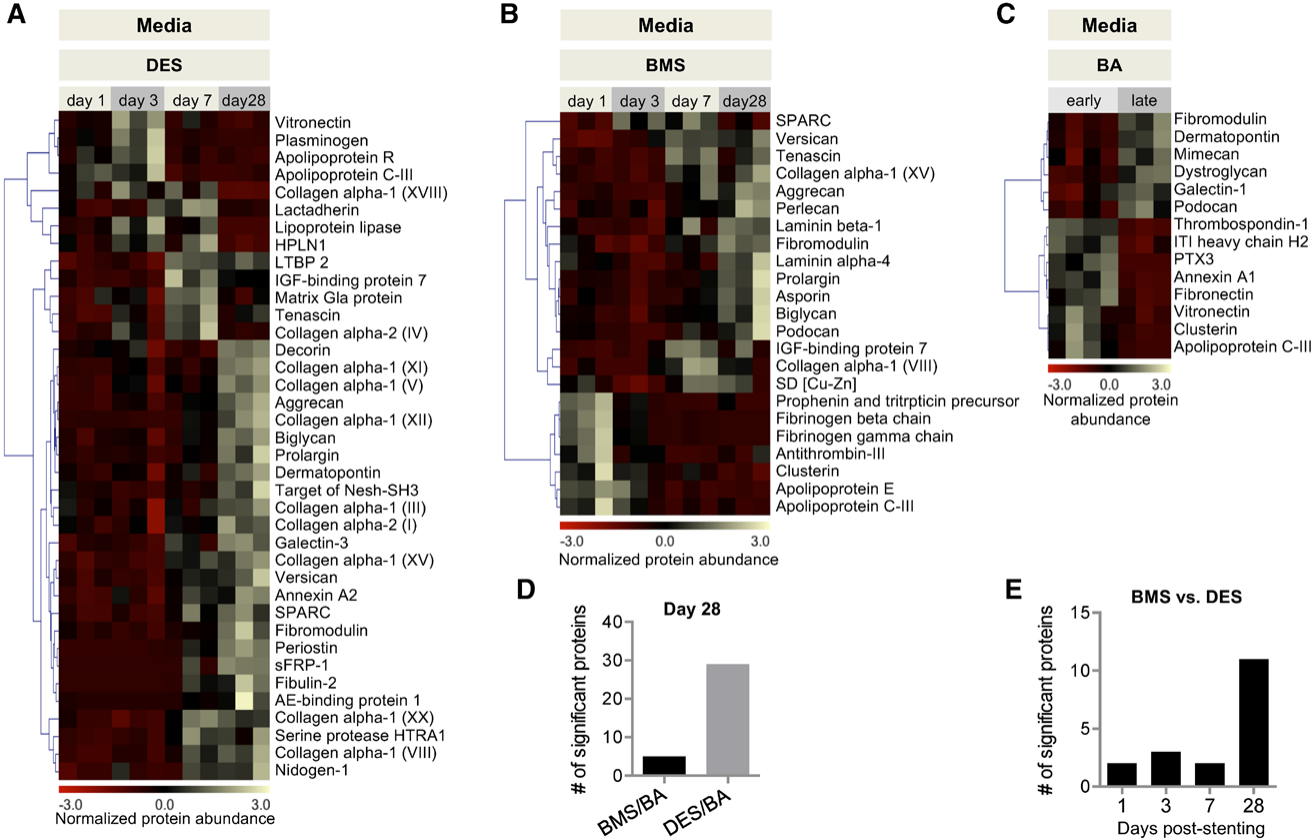
**ECM remodeling in the media.** **A** through **C**, Heat maps of ECM proteins with differential abundance at different time points. BMS/DES: n=3 per time point. *P*<0.05 (1-way ANOVA). BA: n=3–4 per group. *P*<0.05 (*t* test with unequal variance). **D**, Number of significant protein changes in BA late in comparison with BMS and DES at day 28. **E**, Number of proteins with differential abundance between DES versus BMS at day 1, 3, 7, and 28 post–stent implantation. AE indicates adipocyte enhancer; BA, balloon angioplasty; BMS, bare-metal stent; DES, drug-eluting stent; ECM, extracellular matrix; IGF, insulin-like growth factor; ITI heavy chain H2, interalpha-trypsin inhibitor heavy chain H2; LTBP2, latent-transforming growth factor β-binding protein 2; PTX3, pentraxin-related protein PTX3; SD, superoxide dismutase; and sFRP-1, secreted frizzled-related protein 1.

ECM remodeling in response to stent implantation was compared at 4 different time points (Figure 3A and 3B). Coronary arteries subject to BA without stent implantation served as controls (Figure 3C). At an early stage, proteins regulating hemostasis (eg, plasminogen, fibrinogen, antithrombin-III) and inflammation (eg, pentraxin-related protein PTX3, prophenin and tritrpticin precursor) were increased, alongside apolipoproteins found on very low density lipoprotein particles, including apolipoproteins C-III, E, and R.^20^ Proteins with a late response included large aggregating proteoglycans (aggrecan, versican), fibrillar collagens (eg, type I, III, and V), SLRPs (decorin, biglycan, fibromodulin, podocan, asporin), and matricellular proteins (eg, periostin, tenascin, SPARC [secreted protein acidic and rich in cysteine]). In general, fewer proteins changed in the BA group than in stented arteries, and more pronounced changes were seen in DES than in BMS (Figure 3D; Table V in the online-only Data Supplement). Differences in the ECM between DES and BMS became most obvious at day 28 (Figure 3E) and were not revealed by OCT imaging. Thus, DES not only affected cell proliferation, but also the composition of the ECM.

### Comparison of the Media of DES and BMS

**Figure 4.**
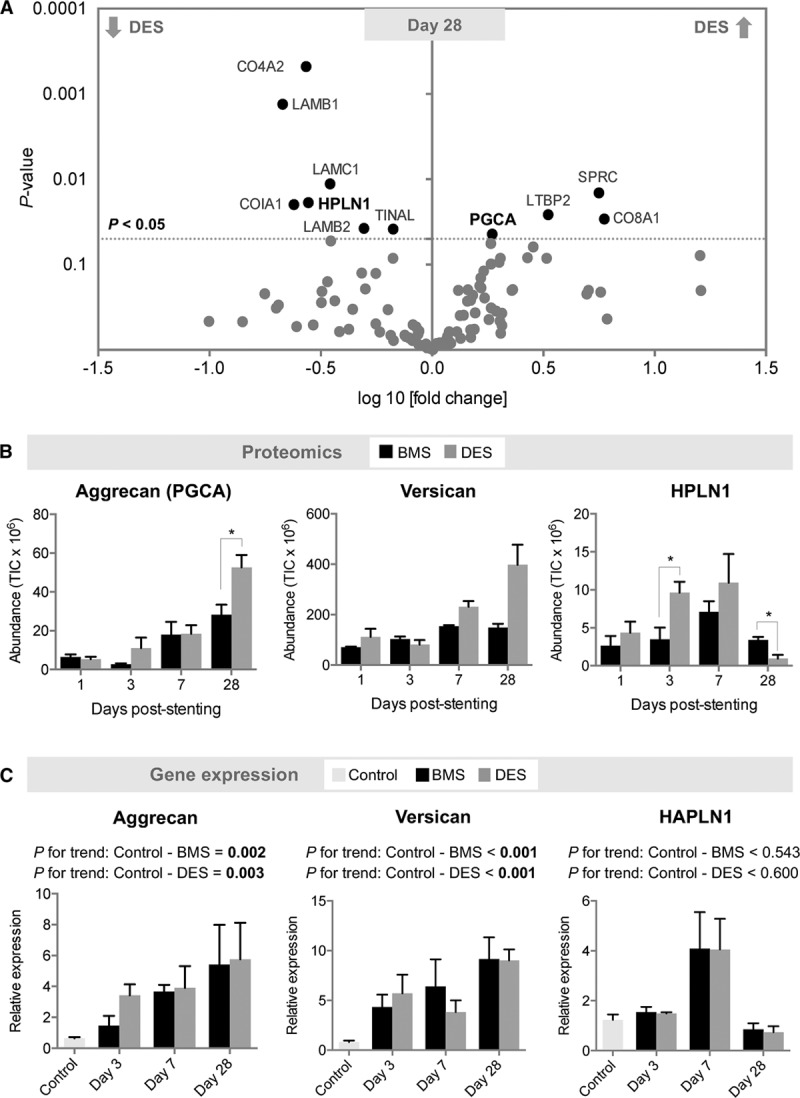
**ECM composition in DES and BMS in the media.** **A**, Volcano plot of differentially expressed proteins between DES and BMS at day 28. n=3 per group (*t* test with unequal variance). **B**, Changes in aggrecan, versican, and HPLN1 protein abundance at day 1, 3, 7, and 28 post–stent implantation. n=3 per group. *P<0.05 (*t* test with unequal variance). **C**, Corresponding gene expression in stented (n=3 per time point) and control unstented coronary arteries (n=6). Gene expression values were normalized to unstented control arteries. Linear regression analysis for *P* value for trend. BMS indicates bare-metal stent; DES, drug-eluting stent; ECM, extracellular matrix; *HAPLN1*, gene name for hyaluronan and proteoglycan link protein 1; HPLN1, hyaluronan and proteoglycan link protein 1; and PGCA, aggrecan; and TIC, total ion current.

At day 28, the antiproliferative effects of DES in comparison with BMS were reflected in a lower abundance of basement membrane proteins such as CO4A2 (collagen α-2 IV), COIA1 (collagen α-1 XVIII), and LAMB1 (laminin β-1), LAMB2 (laminin β-2), and LAMC1 (laminin γ-1 ), as well (Figure 4A; Table VI in the online-only Data Supplement). In contrast, aggrecan was upregulated after stenting, in particular, in DES (Figure 4B). Aggrecan is the major large aggregating proteoglycan in the articular cartilage and interacts with hyaluronic acid. Its rise mirrored the increase of versican, the major large aggregating proteoglycan in the vasculature (Figure 4B). Aggregates of aggrecan and versican with hyaluronic acid are stabilized by HPLN1. HPLN1 was reduced in the media of DES in comparison with BMS (Figure 4B).

### Stent-Induced Changes in Aggrecan and Aggrecanase Expression

Because of its unknown function in the vasculature, aggrecan was selected for further validation. For comparison, we used versican as a structurally and functionally related large proteoglycan. A rise of aggrecan and versican expression after stenting was confirmed at the transcript level (Figure 4C). Consistent with the proteomics findings, there was no equivalent increase in the gene expression of hyaluronan and proteoglycan link protein 1 (*HAPLN1*).

**Figure 5.**
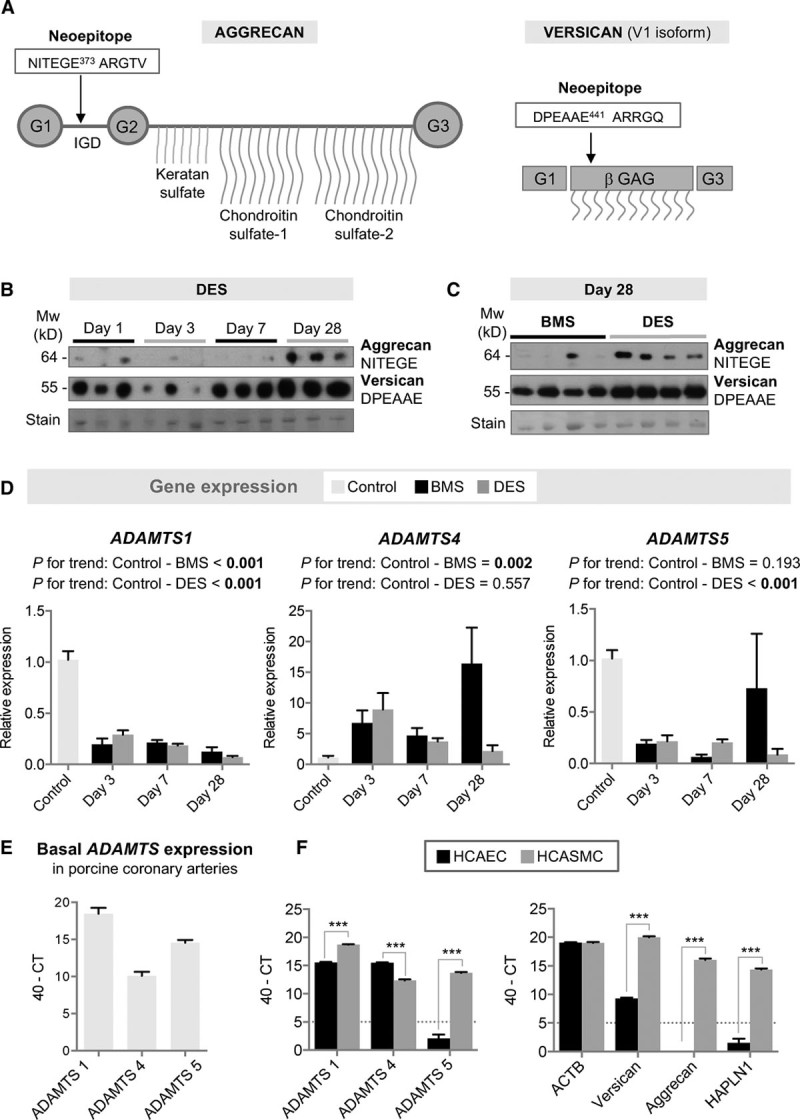
**Aggrecanase expression in the porcine model of stent injury and comparison to human vascular cells.** **A**, The aggrecan neoepitope NITEGE and the versican neoepitope DPEAAE are generated by ADAMTS cleavage. **B**, Aggrecan NITEGE and versican DPEAAE neoepitopes in DES at day 1, 3, 7, and 28 post–stent implantation. n=3 per time point. **C**, Differences between BMS and DES at day 28. n=4 per group. **D**, Gene expression of *ADAMTS1*, -*4*, and -*5* in porcine tissue. n=3 per time point for BMS/DES; n=6 for control coronary arteries. Gene expression values were normalized to unstented control arteries. Linear regression analysis for *P* value for trend. **E**, Basal *ADAMTS* expression levels in porcine coronary arteries. Cycle threshold (CT) values by qPCR are given as 40-CT (higher values indicate higher expression). n=6 per group. **F**, Gene expression of *ADAMTS1, -4*, and *-5* as well as versican, aggrecan, and *HAPLN1* in ECs and SMCs of human coronary arteries. CT values by qPCR are given as 40-CT (higher values indicate higher expression). HCAEC indicate human coronary artery endothelial cell; HCASMC, human coronary artery SMCs; n=triplicates of 3 independent cell passages. ***P<0.001 (*t* test with unequal variance). BMS indicates bare-metal stent; DES, drug-eluting stent; EC, endothelial cell; qPCR, quantitative polymerase chain reaction; and SMC, smooth muscle cell.

Besides gene expression, protein degradation determines the abundance of ECM proteins in the vessel wall. Aggrecanases are members of the ADAMTS (a disintegrin and metalloprotease with thrombospondin motifs) family that can cleave aggrecan and other large aggregating proteoglycans like versican. Thus, we used neoepitope antibodies that only recognize aggrecan and versican after cleavage by members of the ADAMTS family, but not by other proteases like matrix metalloproteinases. For aggrecan, we probed for the signature cleavage at NITEGE^373^-ARGTV in the interglobular domain at the N-terminus, whereas versican fragments were detected by the DPEAAE neoepitope (Figure 5A). The rise in aggrecan was accompanied by increased detectability of the NITEGE neoepitope in DES (Figure 5B, Figure V in the online-only Data Supplement). After an initial loss at day 3, a similar increase of the ADAMTS-generated DPEAAE containing versican fragments was observed (Figure 5B, Figure V in the online-only Data Supplement). At day 28, both were more pronounced in DES than in BMS (Figure 5C, Figure VI in the online-only Data Supplement). These findings demonstrate, for the first time, the contribution of aggrecanases to vascular remodeling on stent implantation.

Because the neoepitope antibodies do not distinguish between the activities of the different aggrecanases and may reflect differences in substrate availability rather than changes in aggrecanolytic activity, we investigated the gene expression of the 3 main ADAMTS enzymes that cleave large aggregating proteoglycans: ADAMTS-1, ADAMTS-4, and ADAMTS-5. After stenting, there was a notable reduction in *ADAMTS1* and *ADAMTS5* gene expression, followed by an increase in *ADAMTS4* (Figure 5D). *ADAMTS1* showed the highest expression levels in porcine coronary arteries (Figure 5E).

### Aggrecan and Aggrecanase Expression in Human Vascular Cells

To relate the findings in the porcine model of stent injury to human vascular cells, we investigated the gene expression of *ADAMTS1, -4*, and -*5* in endothelial cells (ECs) and in SMCs from human coronary arteries (Figure 5F). Human coronary artery ECs express *ADAMTS1* and *ADAMTS4*, but neither *ADAMTS5*, aggrecan, nor *HAPLN1*. In contrast, human coronary artery SMCs express all aggrecanases, including *ADAMTS5*, and aggrecan, versican, and *HAPLN1*, as well. Thus, resident vascular cells can contribute to the aggrecanase activity and aggrecan expression in the arterial wall, with ECs expressing aggrecanases but not aggrecan.

### Aggrecan in the Human Vasculature

**Figure 6.**
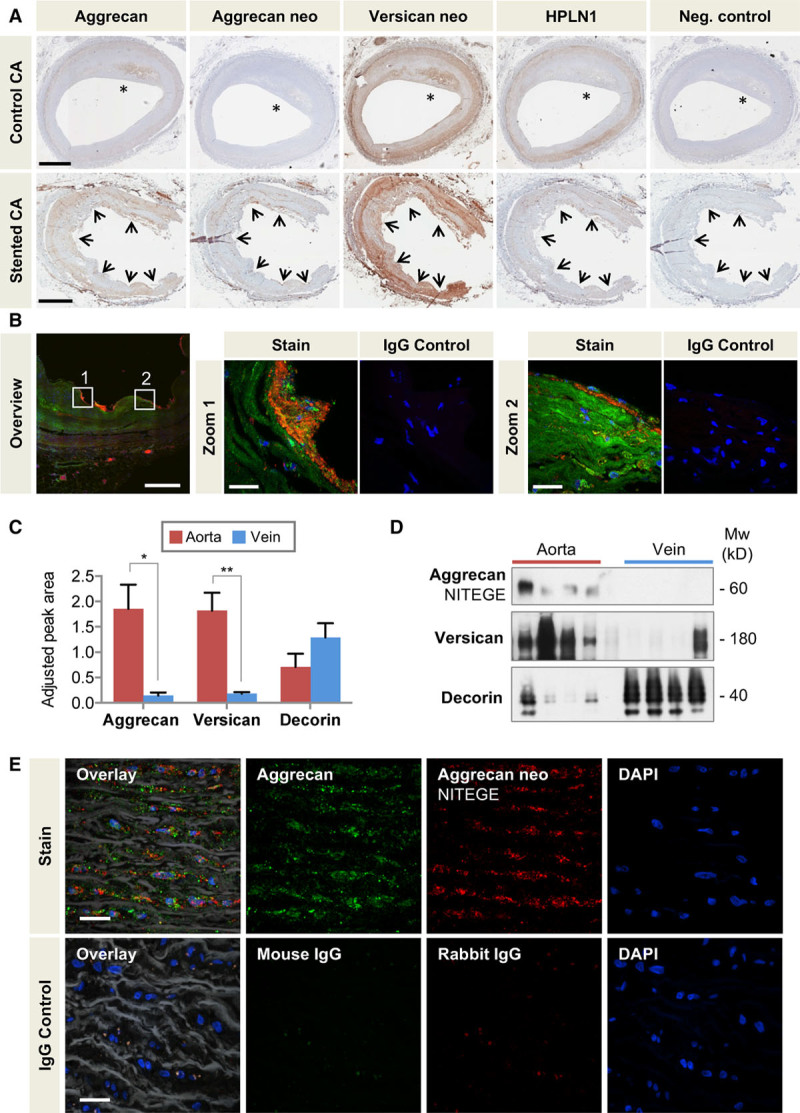
**Aggrecan in the human vasculature.** **A**, Localization of aggrecan, the aggrecan NITEGE, and versican DPEAAE neoepitopes (neo), and HPLN1 in stented and control human coronary arteries with the presence of atherosclerosis (*). Arrows mark the contacts of the stent struts with coronary artery. Scale bars=1 mm. **B**, Colocalization of aggrecan (Alexa 633, displayed in green) and aggrecan NITEGE neoepitope (Alexa 568, displayed in red) in human stented coronary arteries by immunofluorescence. Note the presence of aggrecan fragments at the contacts of the stent struts with the artery and in the subendothelial layer (zoomed-in areas). Overview image 20×, scale bar=500 µm; Zoomed-in areas 60×, scale bar=25 µm. **C**, Adjusted peak area for aggrecan, versican, and decorin in the human thoracic aorta and saphenous veins as determined by targeted proteomics. n=7 per group. *P<0.05, **P<0.01 (*t* test with unequal variance). **D**, Immunoblots for the aggrecan NITEGE neoepitope and versican in the human thoracic aorta and saphenous veins. The SLRP decorin served as a loading control. n=4 per group. **E**, Colocalization of aggrecan (Alexa 633, displayed in green) and aggrecan NITEGE neoepitope (Alexa 568, displayed in red) in human aorta visualized by immunofluorescence. Elastin fibers in white (autofluoresence with 488-nm laser excitation captured in the green emission channel). Control sections stained with isotope IgGs. Magnification 60×, scale bars=20 µm. CA indicates coronary artery; DAPI, 4′,6-diamidino-2-phenylindole; HPLN1, hyaluronan and proteoglycan link protein 1; IgG, immunoglobulin G; and SLRP, small leucine-rich proteoglycan.

To further validate our findings in human tissue, we used immunohistochemistry to localize aggrecan and aggrecan cleavage in stented human coronary arteries (Figure 6A). Coronary arteries with atherosclerotic plaques but without stents served as a reference control. Aggrecan and HPLN1 colocalized in the media of control arteries. In stented coronary arteries, staining for the aggrecan NITEGE neoepitope was observed predominantly below the periluminal cellular layer and at the contacts of the stent struts with the coronary artery (Figure 6B, Figure VII in the online-only Data Supplement). This was also supported by the finding that human coronary artery ECs express *ADAMTS1* and *ADAMTS4* (Figure 5F). To further investigate aggrecan in the human vasculature, we compared specimens of human saphenous veins and human thoracic aorta. Targeted proteomics revealed that, similar to versican, aggrecan was more abundant in the aorta than in veins (Figure 6C, Table VII in the online-only Data Supplement). The SLRP decorin served as a control. Likewise, the ADAMTS-specific aggrecan NITEGE neoepitope was detectable in the aorta (Figure 6D, Figure VIII in the online-only Data Supplement). Aggrecan and ADAMTS-induced aggrecan cleavage was seen throughout the media of the human aorta as confirmed by immunofluorescence staining (Figure 6E).

### Aggrecan and Aggrecanases in Mice

**Figure 7.**
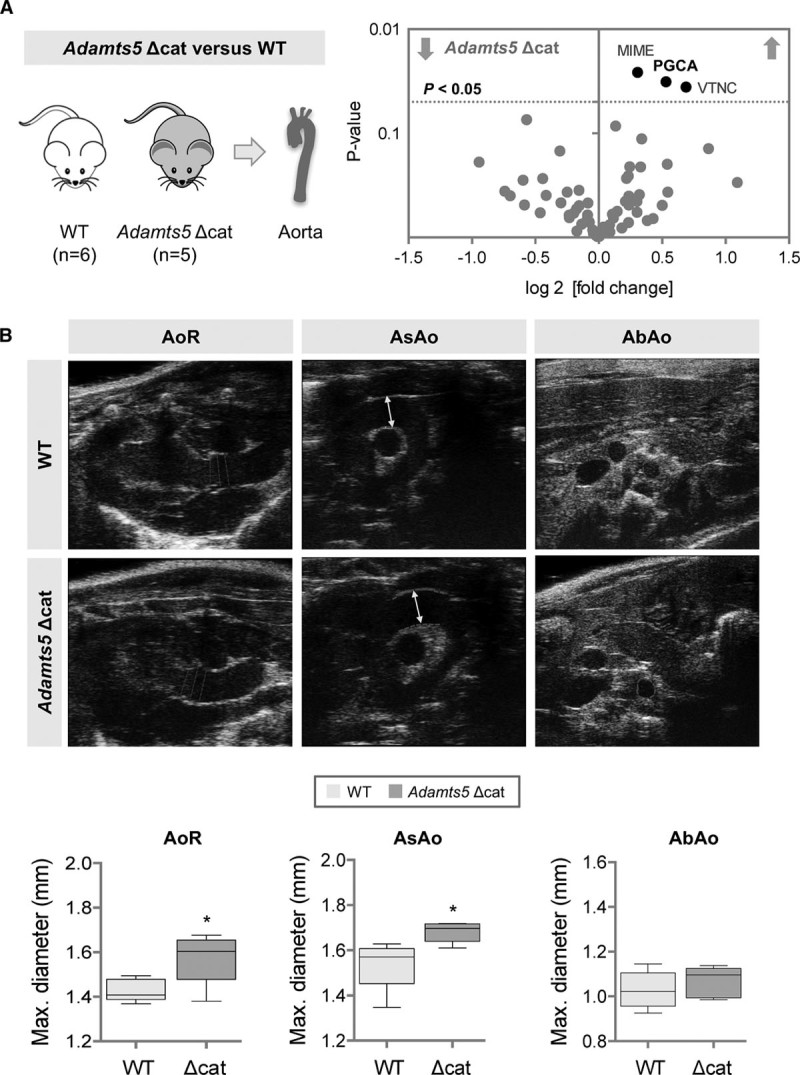
**Aggrecan and aggrecanases in the murine aorta.** **A**, Volcano plot of differentially expressed proteins between the aorta of wild-type littermate controls (WT) and mice lacking the catalytic domain of ADAMTS-5 (*Adamts5* Δcat). n=6 (WT), n=5 (*Adamts5* Δcat), *t* test with unequal variance. PGCA indicates aggrecan; MIME, mimecan; and VTNC, vitronectin. **B**, Aortic diameters of aortic ring (AoR), ascending aorta (AsAo), and abdominal aorta (AbAo) as measured by ultrasonography. Representative ultrasound images are shown, values are represented as box-and-whisker plots. n=5 per group *P<0.05 (*t* test with unequal variance).

ADAMTS-5 is the main aggrecan-degrading enzyme in mice.^21^ To examine the effects of ADAMTS-5 on the vasculature, we used proteomics to compare the ECM of aortas from mice lacking the catalytic domain of ADAMTS-5^21^ with aortas from littermate controls (Table VIII in the online-only Data Supplement). Loss of ADAMTS-5 activity resulted in an accumulation of aggrecan (Figure 7A) confirming the importance of aggrecanases in regulating the abundance of aggrecan in the vessel wall. These structural changes in the aortic wall were accompanied by an increase in the diameter of the thoracic, but not of the abdominal aorta (Figure 7B). It is noteworthy that cardiac output as measured by cardiac MRI and blood pressure were not significantly different from wild-type controls (Figures IX and X in the online-only Data Supplement). This genetic mouse model confirms the importance of aggrecanases in regulating the abundance of aggrecan in the vessel wall and suggests a link between aggrecanase activity and aortic diameter.

**Figure 8.**
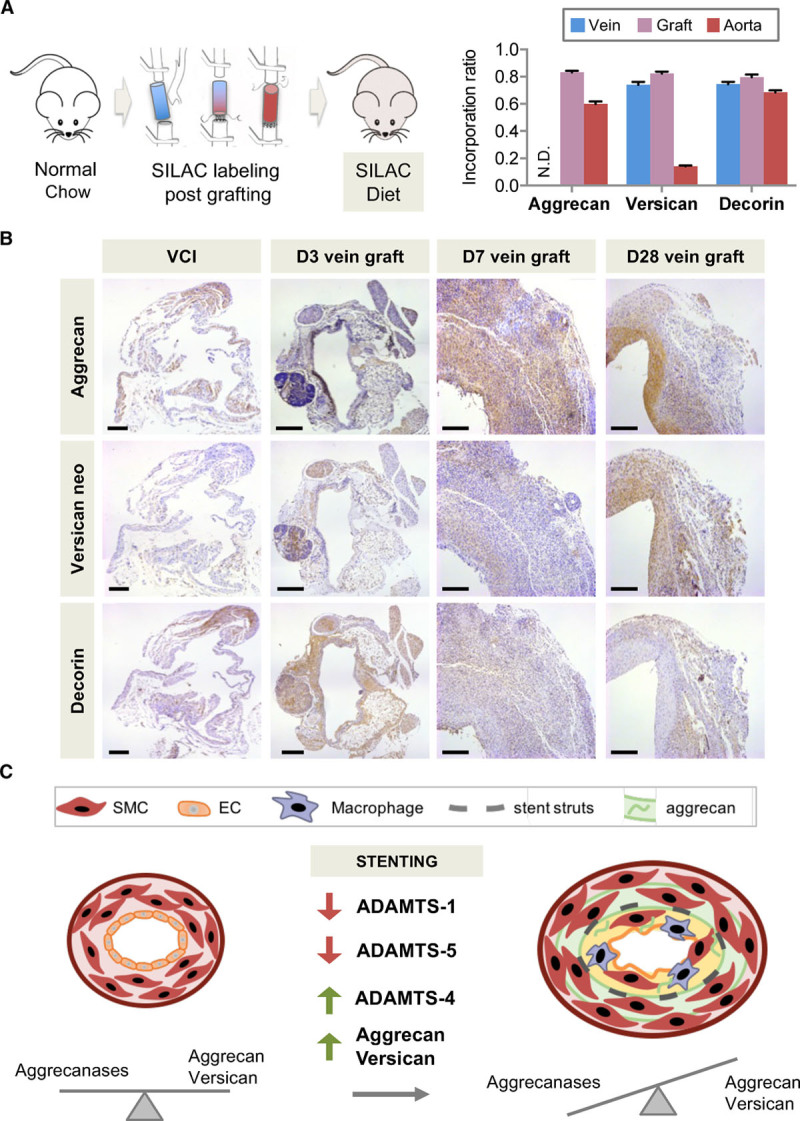
**Aggrecan in vascular remodeling.** **A**, The isogenic venae cavae were grafted to carotid arteries of mice fed a diet with stable isotope-labeled amino acids. Incorporation ratios for aggrecan, versican, and decorin in interposition grafts, vena cava inferior, and aortas. n=8 to 9 per group. N.D. indicates not detectable. **B**, Immunostaining for aggrecan, the versican DPEAAE neoepitope, and decorin. Magnification 10×, scale bars=200 µm. **C**, Summary. Changes in ADAMTS expression accompany the increase of the large aggregating proteoglycans aggrecan and versican post-stenting. EC indicates endothelial cell; SILAC, stable isotope labeling with amino acids in cell culture; and SMC, smooth muscle cell.

The higher abundance of aggrecan in stented porcine coronary arteries and human aortas in comparison with veins is suggestive of mechanical stretch inducing aggrecan expression in the vasculature. To explore this potential mechanism, veins were grafted into the carotid arteries of mice.^22^ After grafting, mice were fed a diet of stable isotope-labeled amino acids for 28 days to label newly synthesized proteins (Figure 8A). With the use of targeted LC-MS/MS analysis (Table IX in the online-only Data Supplemental), the incorporation of the stable isotope-labeled amino acids was compared between ECM proteins in veins, vein grafts, and arteries. Sixty percent of aggrecan peptides were found to be labeled in the murine aorta, demonstrating active synthesis of this proteoglycan in the arterial wall. In contrast, labeled aggrecan peptides were undetectable in veins. The incorporation ratio in vein grafts, however, was comparable with the aorta. Thus, aggrecan synthesis was inducible in veins by exposure to arterial blood pressure. Representative histological sections are shown in Figure 8B, where aggrecan was markedly elevated after grafting. The upregulation coincided with cleavage of versican as indicated by staining for the ADAMTS-specific DPEAAE neoepitope. Thus, besides their established role in cartilage, aggrecan and aggrecanases are important contributors to ECM remodeling in the vasculature on stenting and mechanical stretch (Figure 8C).

## Discussion

This is the first proteomics analysis to profile the ECM remodeling of stented coronary arteries and to compare the effects of DES versus BMS on the vascular ECM. Diverging from the traditional focus on collagens and matrix metalloproteinases, this study highlights the contribution of aggrecan and aggrecanases to ECM remodeling after stent implantation. Findings in the large-animal model were validated in human specimens and followed-up in 2 small animal models. Thus far, little is known about the role of aggrecan in the vasculature and the stimuli that induce its expression.^23^ We demonstrate that aggrecan is more abundant in arteries than in veins, that the synthesis of aggrecan is inducible by grafting veins to arteries, and that aggrecan abundance is influenced by aggrecanase activity in the arterial wall.

### ECM Changes in the Neointima

The vascular ECM plays a critical role in providing structural support to the vessel wall and influences cell behavior and signaling. Our proteomics comparison of the neointima revealed the following findings: First, a more organized and structured ECM assembly in the neointima forming over BMS in comparison with DES. Presumably, ECM organization occurs at an earlier stage in arteries treated with BMS than in DES. Second, an upregulation of proteins associated with the regulation of calcification in neointimal lesions of arteries treated with DES. The upregulation of these proteins may be a protective response as demonstrated by the presence of MGP and SPP24 in human control arteries. MGP is thought to act as an inhibitor of calcification.^24^ Similarly, SPP24 is not osteogenic but binds to and affects the activity of bone morphogenetic protein 2.^25^ Indeed, calcification assays in human arterial SCMs demonstrated an inhibition of calcification under everolimus treatment. Everolimus is an inhibitor of the mechanistic target of rapamycin (mTOR). Further studies will need to clarify whether this observation is directly related to the observed ECM protein changes on drug treatment or other drug effects. Drug effects could induce a phenotypic change of SMCs. This may also explain why MGP and SPP24 appear increased in normal vessels versus stented human vessels. Finally, we observed chondroadherin in DES-stented arteries. This SLRP is mainly expressed in cartilaginous tissue^26^ and has been previously associated with disc degeneration,^27^ but its expression in the vasculature has never been reported at the protein level. Yet, there is evidence for an association of chondroadherin with vascular pathology, because its transcription was found to be specifically induced in atherosclerotic plaques of femoral arteries.^28^ It remains to be elucidated if these proteins might provide a link to the increased incidence or accelerated course of de novo atherosclerosis within DES-stented vessels.^29^

### Early Changes on Vascular Injury

Our discovery-based proteomics comparison allowed an analysis of ECM proteins during early and late stages of vascular healing in response to stents. The early response to injury was independent of the applied stent type. Some changes were also observed after BA alone without stent insertion, indicating a response to vascular injury rather than stenting. Three different functional classes were increased: first, proteins involved in thrombosis such as fibrinogen, plasminogen, antithrombin III, and thrombospondin, which is in line with findings in histopathologic studies of platelet activation and thrombosis formation during the early stage of vascular healing^30^; second, inflammatory proteins associated with innate immunity, such as PTX3 and prophenin and tritrpticin, implicating the recruitment of inflammatory cells to the site of vascular injury. Lactadherin, for example, contributes to phagocytic removal of apoptotic cells^31^ and shows a peak expression at 7 days post–stent insertion in the DES group. Finally, various apolipoproteins, such as apolipoproteins E, C-III, and R and also lipoprotein lipase were retained. It is noteworthy that these are very low density lipoprotein–associated apolipoproteins.^20^ We have recently observed that plasma levels of very low density lipoprotein–associated apolipoproteins predict cardiovascular events^32^ and that these apolipoproteins are also present in human atherosclerotic plaques.^33^

### ECM Changes in the Media

ECM changes were observed predominantly after 28 days, which is likely explained by the time it takes for SMCs to lay down enough new ECM. A significant increase at day 28 was seen in fibrillar collagens, such as type I, III, and V, and matricellular proteins (periostin, tenascin, SPARC) and SLRPs like decorin, biglycan, fibromodulin, podocan, and asporin, as well. These are important regulatory proteins and involved in a variety of cellular functions such as collagen fibril assembly, inflammation, cell proliferation, adhesion, and migration, and fibrosis, as well.^34^ In general, more proteins showed a significant change in the DES group than in the BMS group, with the differences being more pronounced at later stages. In DES, a uniform downregulation of basement membrane proteins, indicative of the reduced cellularity, was accompanied by an increase in aggrecan, also known as cartilage-specific proteoglycan core protein.

### Aggrecan in the Vasculature

Aggrecan is well studied in cartilage, but its expression and function in the vasculature has only recently begun to be appreciated. It is the major proteoglycan in cartilage^35,36^ and characterized by its ability to bind hyaluronan, a large carbohydrate polymer, to form even larger aggregates.^37^ Between its G2 and G3 domain aggrecan carries numerous glycosaminoglycans, namely keratan sulfate and chondroitin sulfate chains, giving the protein an enormous amount of fixed negative charges (Figure 5A). These highly negatively charged glycosaminoglycans provide the basis for the viscoelastic properties of cartilage.

Because of its water-attracting property that confers resistance to compression, aggrecan may be part of an adaptive response of the vasculature to absorb mechanical forces. In addition, proteoglycans present growth factors and cytokines to the surrounding tissue,^38^ and their interactions with other ECM components modulate a wide range of cellular responses, including inflammation.^39^ It has been suggested that the lipoprotein binding to negatively charged glycosaminoglycan side chains might contribute to atherosclerosis.^23,40^ Aggrecan expression was demonstrated in advanced lesions of atherosclerotic aortas of ApoE/LDLr-deficient mice by immunohistochemistry, further supporting that cartilage-associated ECM proteins may be involved in the pathogenesis of atherosclerosis.^41^ Similarly, we observed staining for aggrecan in the atherosclerotic lesion of the human control coronary artery (Figure 6A, *indicates plaque location). It is noteworthy that a previous proteomics analysis discovered aggrecan in vascular intimal hyperplasia.^42^ Our proteomics study provides the first evidence for an upregulation of aggrecan after stenting and on grafting veins from a low- to a high-pressure environment. Future studies are needed to investigate whether aggrecan or its cleavage products may contribute to the more frequent and accelerated onset of neoatherosclerosis in DES.^43^ Kumar et al^44^ have recently reported that reduced aggrecan cleavage was associated with decreased macrophage numbers and less atherosclerosis in *Adamts4*^–/–^ *ApoE*^–/–^ mice.

### Aggrecanases in the Vasculature

Besides aggrecan, the aggrecan NITEGE neoepitope was detected in porcine coronary arteries on stenting. This aggrecan neoepitope is generated on digestion by aggrecanases of the ADAMTS family, but not by other proteases. Because aggrecan is not abundant in uninjured vessels (Figure 4B), the NITEGE neoepitope predominantly reflects differences in substrate availability (Figure 5B). Instead, the versican DPEAAE neoepitope reflects the loss of aggrecanolytic activity at day 3 post-stenting (Figure 5B). The neoepitope antibodies do not distinguish between the cleavage products of ADAMTS-1, ADAMTS-4, and ADAMTS-5 activity. *ADAMTS1, ADAMTS4*, and *ADAMTS5*, however, showed a notable shift in gene expression in stented porcine coronary arteries. Expression of *ADAMTS1* and *ADAMTS5* was markedly reduced on stent implantation. Instead, *ADAMTS4* was induced, in particular, in BMS. Human coronary artery ECs only express *ADAMTS1* and *ADAMTS4*, whereas human coronary artery SMCs express *ADAMTS1*, *ADAMTS4*, and *ADAMTS5*, and aggrecan, as well. Thus, the loss of arterial ECs on stenting could contribute to the initial loss of aggrecanase expression in the vessel wall.^45^ Moreover, the effects of DES might facilitate the accumulation of large aggregating proteoglycans in the vessel wall. Treatment of human coronary artery SMCs with everolimus reduced gene expression of *ADAMTS4*, whereas the expression levels of *ADAMTS1* remained unchanged (data not shown). On vascular injury, however, inflammatory cells are likely to contribute to the ADAMTS-4 activity in the vessel wall.^44^

Aggrecan and its ADAMTS-cleaved fragments were also colocalized in the media of the human thoracic aorta. Aggrecanase activity regulates the abundance of aggrecan in the vessel wall as evidenced by the buildup of aggrecan in the aorta of mice lacking the catalytic domain of ADAMTS-5. Their vascular phenotype closely resembled recent observations in *Adamts-1* heterozygous mice.^46^ In *Adamts-1*^+/–^ mice, thoracic aortic aneurysm formation and dissection were induced by an increase in inducible nitric oxide synthase and medial degeneration. In our analysis, loss of ADAMTS-5 activity resulted in thoracic aortic dilation with a buildup of aggrecan. This finding extends our previous report on the catabolic properties of ADAMTS-5 on vascular proteoglycans and its ability to alter proteoglycan-mediated lipoprotein retention in a mouse model of atherosclerosis.^23^

### Strengths and Limitations

A particular strength of our study is the combination of proteomics with a clinically relevant large-animal model of human disease.^15^ In the porcine model, peak neointima growth is observed at 28 days after stent deployment corresponding to 6 months in humans. Few changes occur beyond this time point, with the exception of slight neointima thinning later on.^14^ The pigs, however, are young and free of atherosclerosis. A caveat of working with porcine tissue is the limited availability of antibodies, and proteomics was key for a comprehensive protein analysis without constraints imposed by antibodies.

## Conclusions

The effects of DES go beyond inhibition of SMC proliferation, but have a wider impact on vascular remodeling by altering the composition of the ECM. The antiproliferative effect of DES was evident by a reduction in basement membrane proteins. Moreover, the decreased cellularity was accompanied by increased production of large aggregating proteoglycans like aggrecan and versican. Aggrecan and aggrecanases are integral components during ECM remodeling after stenting. A summary of our findings is provided in Figure 8C.

## Acknowledgments

Stents were supplied free of charge by Abbott Vascular. Fluorescent microscopic images were acquired at the Nikon Imaging Center at King’s College London. The authors acknowledge the technical contribution of N. Catibog and Dr M. Chong. The authors thank Dr Pechlaner for his advice on the statistical analysis.

## Sources of Funding

Dr Suna was supported by a PhD studentship from King’s British Heart Foundation (BHF) Centre. Prof Wojakowski received funding from RMT foundation. Dr White is supported by BHF CH95/001 and the National Institute for Health Research (NIHR) Bristol Biomedical Research Unit in Cardiovascular Medicine. Prof Mayr is a BHF Chair Holder (CH/16/3/32406) with BHF program grant support (RG/16/14/32397) and supported by the NIHR Biomedical Research Center based at Guy’s and St Thomas’ National Health Service Foundation Trust and King’s College London in partnership with King’s College Hospital. Prof. Mayr is also supported by an excellence initiative (Competence Centers for Excellent Technologies [COMET]) of the Austrian Research Promotion Agency (FFG): Research Center of Excellence in Vascular Ageing—Tyrol, VASCage (K-Project Nr. 843536) funded by the Federal Ministry for Transport, Innovation and Technology (BMVIT), Federal Ministry of Science, Research and Economy (BMWFW), the Wirtschaftsagentur Wien, and the Standortagentur Tirol.

## Disclosures

Dr Hill is part of the Consultant Advisory Board at St Jude Medical.

## Supplementary Material
