# Supplementary material for: Extracellular Matrix Proteomics Reveals Interplay of Aggrecan and Aggrecanases in Vascular Remodeling of Stented Coronary Arteries
Source: Circulation. 2018 Jan 8;137(2):166–83. doi: 10.1161/CIRCULATIONAHA.116.023381 (PMC5757669; doi:10.1161/CIRCULATIONAHA.116.023381)
Supplement: Supplementary file 1 [file cir-137-166-s001.pdf]

SUPPLEMENTAL MATERIAL

Extracellular Matrix Proteomics Reveals Interplay of Aggrecan and Aggrecanases in Vascular Remodeling of Stented Coronary Arteries

Gonca Suna, MD, PhD[1]; Wojciech Wojakowski, MD[2]; Marc Lynch, MSc[1]; Javier Barallobre-Barreiro, PhD[1]; Xiaoke Yin, PhD[1]; Ursula Mayr, MD[1]; Ferheen Baig, MSc[1]; Ruifang Lu, PhD[1]; Marika Fava, PhD[1]; Robert Hayward, BSc[1]; Chris Molenaar, PhD[1]; Stephen J. White, PhD[3]; Tomasz Roleder, MD[2]; Krzysztof P. Milewski, MD[4]; Pawel Gasior, MD[2]; Piotr P. Buszman, MD[4]; Pawel Buszman, MD[4], Marjan Jahangiri, MD[5]; Cathy Shanahan, PhD[1]; Jonathan Hill, MD[6]; Manuel Mayr, MD, PhD[1]

[1]King's British Heart Foundation Centre, King's College London, London, UK

[2]3rd Division of Cardiology, Medical University of Silesia, Katowice, Poland

[3]Healthcare Science Research Centre, Manchester Metropolitan University, Manchester, UK

[4]Centre for Cardiovascular Research and Development, American Heart of Poland, Katowice, Poland

[5]St. George's Vascular Institute, St George's Healthcare NHS Trust, London, United Kingdom

[6]King's College Hospital and King's Health Partners Academic Health Sciences, London, United Kingdom.

Address for Correspondence:

Prof. Manuel Mayr, MD, PhD, King's British Heart Foundation Centre, King's College London, 125 Coldharbour Lane, London SE5 NU, UK, Phone: +44 (0) 207848 5446

Fax: +44 (0) 20 7848 5298, Email: manuel.mayr@kcl.ac.uk

Twitter: https://twitter.com/Vascular_Prot

SUPPLEMENTAL METHODS

OCT Image Analysis in Pigs. OCT imaging was performed at stent implantation and at follow-up using the ILUMIEN OPTIS imaging system (St. Jude Medical, US). The OCT probe (mid marker of the OCT Dragonfly catheter) was positioned 5 mm distally to the analysed stent. All OCT imaging was performed using automated pullback triggered by hand injection of contrast. Every 1 mm of the stent was scrutinized by OCT to assess minimal lumen area, stent struts apposition, stent struts coverage and neointimal volume. Qualitative analysis of neointima was performed classifying the neointimal structure into heterogeneous, layered or homogenous with peristrut attenuation or ring.[1] If peristrut attenuation was visible in the whole circumference of the stent at the single OCT cross-sectional frame, the peristrut ring was recognized. The data was analysed using the OCT image analysing system, CAAS IntraVascular (version 1.1, Pie Medical Imaging).

Deglycosylation of ECM Extracts. GuHCl was removed by ethanol precipitation and the proteins were deglycosylated using deglycosylation enzymes. The deglycosylation enzymes included: chondroitinase ABC (1:100), keratanase (1:500) and heparinase II (1:500) (all from Sigma-Aldrich) for the removal of GAG side chains; 2 different debranching enzymes including α2-3,6,8,9-Neuraminidase (1:200) and ß-N-acetylglucosaminidase (1:200) as well as O-glycosidase for the removal of the remaining O-linked sugars (1:200) (all from Millipore). After 24 h incubation samples were speed-vac dried. PNGase F (1:200, from Millipore) was added together with ^{18}O labelled water (Sigma-Aldrich) for another 48 h of incubation.

Generation of Custom-made Porcine ECM Protein Database. To achieve the best peptide sequence coverage by LC-MS/MS, and identify as many proteins as possible, a search database containing a comprehensive list of porcine ECM proteins was generated. Previously reported cardiovascular ECM proteins identified in human, porcine and murine cardiovascular tissues[2-4] were searched for their porcine sequences in the Uniprot protein database. Porcine sequences of many of these proteins were found and retrieved from Uniprot either as annotated proteins or after blasting the human sequence against non-annotated,

uncharacterized porcine proteins. The remaining ECM proteins with no matching sequences in Uniprot were deduced from public nucleotide databases (including mRNA annotated sequences and expressed sequence tags [EST]) using tBLASTtn. Exclusively the best matches (≥ 90% identity) were assigned to a protein sequence and retrieved for the database. The final database contained a total of 270 manually included porcine ECM protein sequences. All canonical human sequences were used as background in order to obtain a database suitable for search algorithms (i.e. Mascot). To avoid redundancies all human ECM proteins were manually removed and replaced by their porcine counterparts.

Human Tissue. All procedures involving use of human tissues were approved by a local Research Ethics Committee (London, United Kingdom, Wandsworth research ethics committee approval REC number 08/H0803/257). Normal saphenous vein specimens were obtained by surgical resection in consented patients undergoing coronary artery bypass surgeries; the segment of the saphenous vein near the knee level was collected for analysis from each patient. Control aortic samples from consented patients without connective tissue disorder were obtained upon aortotomy performed during routine aortic valve replacement from positions of the ascending aorta that were free of macroscopically evident vascular pathology. All samples were snap frozen following surgical removal. There were no known potential confounders during sampling of clinical specimens and tissues.

Immunoblot Analysis. Porcine samples: 10 µg of deglycosylated GuHCl extracts of stented porcine coronary arteries (n=3 first cohort; n=4 second cohort day 28) were denatured at 95 °C for 5 min and reduced with sample buffer (0.1 mol/l Tris, pH 6.8, 40% glycerol, 2% SDS, 2% beta-mercaptoethanol and 0.02% bromophenol blue) and separated by SDS-PAGE. Subsequently proteins were blotted to a nitrocellulose membrane, which was blocked in 5% milk and incubated with primary antibodies to the aggrecan NITEGE neoepitope (Thermo, AF-PA1-1746) and the versican DPEAAE neoepitope (Abcam, ab19345) over night. After 3 washes (15 min each) with PBS-Tween, the membranes were treated with appropriate HRP-conjugated light chain-specific detection antibody (1:5000, Jackson Immunoresearch) for 1 h. The membranes were washed again 3 times and developed using enhanced

chemiluminscence (ECL, GE Healthcare) on a Xograph processor in 30 sec increments. ImageJ software was used for densitometric quantification. _Human samples_: Same protocol was applied. 7 µg of GuHCl extracts of human thoracic aorta and saphenous vein were loaded per lane. Antibodies against the aggrecan NITEGE neoepitope (Thermo, AF-PA1-1746), versican (Santa Cruz, sc-25831) and decorin (Thermo Fisher PA5-19151) were used.

Cell Culture. _Endothelial cells:_ Human coronary artery endothelial cells were obtained from PromoCell (C-12222) and cultured on 0.04% gelatine in appropriate Endothelial Cell Growth Medium (PromoCell C-22020) at 37 °C in a humidified atmosphere of 95% air/5% CO_2. Cells were washed with cold PBS twice and scraped off the surface with Qiazol. RNA was extracted as described below and reversely transcribed using SuperScript® VILO MasterMix (Life Technologies). The experiments were carried out with cells at passage 4-9. _Smooth muscle cells_: Human coronary artery smooth muscle cells (HCASMCs) were obtained from PromoCell (C-12511) and grown in M199 medium (Gibco™) on 0.04% gelatine. For experiments cell passages 7-11 were used.

Calcification Assay. Human aortic SMCs were cultured in M199 supplemented with 5% fetal bovine serum and treated with everolimus 20 nM or equivalent concentration of DMSO as vector control. Everolimus was a kind gift from Novartis, Switzerland and was shipped under a material transfer agreement with King's College London. To induce mineralization the medium was supplemented with calcium (2.7 mM) and phosphate (2.5 mM).[5] Calcification was visualized after fixation with 4% formaldehyde using Alizarin red (2% weight/volume, pH 4.2) and quantified spectrophotometrically using the o-Cresolphthalein complexone assay after 8 days of Ca/P treatment +/- everolimus. Calcium concentrations were normalized to protein concentrations determined by the Biorad DC protein assay. Each experiment was carried out in triplicates and cells were used at passages between 10-12.

Real-time Polymerase Chain Reaction (qPCR). RNA from the porcine tissue was extracted using the miRNeasy Mini kit (Qiagen) according to the manufacturer's instructions. For gene expression analysis, porcine RNA was reversely transcribed using SuperScript® VILO MasterMix (Life Technologies). Taqman® assays were used to assess the expression

of individual target genes. Diluted reverse transcription products were combined with TaqMan® expression (Applied Biosystems) and TaqMan® Universal PCR Master Mix No AmpErease® UNG (2x) to a final volume of 5µl. For ADAMTS-1, -4 and -5 expression analysis in porcine tissue samples, SYBRgreen primers were designed and the reaction volume was set up with SYBR Select Master Mix (2x):

ADAMTS1: fwd CGTGAACAAGACCGACAAGA/ rev AACTCCTCCACCACACGTTC;
ADAMTS4: fwd CCCCATGTGCAACGTCAAG/ rev AGTCTCCACAAATCTGCTCAGTGA;
ADAMTS5: fwd TCACGAAATTGGACATCTGC/ rev CTGGTCAGGATGGAGGACAT.
ACTB: fwd TCTGGCACCACACCTTCT/ rev GATCTGGGTCATCTTCTCAC).

The qPCR reaction was performed in a ViiA7 qPCR instrument (ThermoFisher Scientific) under following conditions: 95 °C for 10 min, followed by 40 cycles of 95 °C for 15sec and 60 °C for 1 min. Samples were normalized to the expression of β-actin (ACTB) for gene expression targets. Target relative amounts were quantified using the $2^{-\Delta\Delta CT}$ method.[6]

Vein Graft Surgery in Mice. An established mouse model of venous bypass graft was used by grafting *isogeneic venae cavae* to common carotid arteries of female C57BL/6J mice (Jackson laboratories), as described previously.[7] Briefly, mice were anaesthetised by intraperitoneal injection of ketamine (75 mg/kg) and medetomidine HCL (1 mg/kg). A segment (approx. 1 cm) of vena cava from a donor animal was grafted end-to-end to carotid arteries using a cuff technique. Grafts and vena cava were harvested at time point 3, 7 and 28 days post-surgery for histological characterization. Other mice were fed a SILAC (stable isotope labeling with amino acids in cell culture) diet containing heavy lysine *ad libitum* and had access to water. Stable isotope-labelled mouse feed was obtained from Silantes (Munich, Germany). Mice were euthanized by anesthetic overdose 28 days postoperatively. The vein grafts were harvested by cutting the implanted segments from the native vessels at the cuff end together with the vena cava and the aorta. The vessels were processed using the ECM extraction method as described above. GuHCl extracts were subject to tryptic digests and used for targeted proteomics analysis as described in the following paragraph.

Targeted Proteomic Analysis.

SILAC-fed mice tissue: Proteotypic peptides were scheduled using the retention time obtained from untargeted experiments with same HPLC configuration and eluting gradient. All proteotypic peptides were lysine (K)-terminated to allow quantification of SILAC incorporation. Skyline software (MacCoss Lab, University of Washington, Seattle) was used first to predict collision energies and optimize retention times, and second to quantify peak areas for MS2 ions after Parallel Reaction Monitoring (PRM). The identity of a specific peptide was confirmed by the presence of multiple transitions at the same retention time. Retention time windows were set +/- 4 min. All peaks were manually reviewed and integrated. A mass tolerance of 5ppm was used as a minimum requirement for fragment ions. SILAC incorporation rates were derived from the proportion between ions derived from peptides containing either heavy or light lysines (i.e. Lys152 and Lys146) using the following formula: Incorporation rate = [Heavy] / [Heavy + Light]. The intensities of heavy and light peptides were calculated using the peak areas after MS2. The following peptides were used for targeted proteomics in mice tissue: aggrecan GDPETSVSGVGDDFSGLPSGK (G1172 – K1192), TVYLYPN[+3]QTGLPDPLSK (T661 – K677); versican VSVPTHPDDVGDASLTMVK (V101 – K119); decorin DLHTLILVNNK (D101 – K111), NSGIENGAFQGLK (N183 – K195), and VVQC[+57]SDLGLDK (V59 – K69).

Human vascular tissue: For targeted proteomics in human aorta and veins same methodology was applied except the SILAC labeling. Following peptides were used: aggrecan C[+57]GGNLLGVR (C318 - R326), versican LATVGELQAAWR (L277 - R288), decorin NLHALILVNNK (N106 - K116).

Immunofluorescence Staining in Human Vessels. Vessels were processed as described for immunohistochemistry. Sections of 5 µm (aorta) or 3 µm (coronary artery) were incubated with 0.5 unit/ml chondroitinase ABC (Sigma C3667) for 1 h at 37 °C. After blocking with 10% donkey serum in PBS for 1 h, sections were co-incubated with primary antibodies to aggrecan (1:10; Abcam, ab3778) and the aggrecan NITEGE neoepitope (1:200; Thermo, AF-PA11746) as well as matched isotope IgGs for negative controls. Secondary antibodies (Life Technology) were applied as indicated. Cell nuclei were stained with DAPI (Life Technology).

Sections were visualized with a 20x CFI S Plan Fluor ELWD ADM objective or 60x Plan Apo VC NA 1.40 Nikon using an inverted Nikon NI-E microscope equipped with a Yokogawa CSU-X1 Spinning disk confocal unit and an Andor iXon 3 EM-CCD camera. Images were acquired using NIS-elements 4.0 software, and represent a maximum projection image of a Z-stack of 0.5 µm steps compassing 9 µm.

Immunohistochemistry in Murine Vein Grafts. For histological analysis, mice tissues were fixed overnight with 10% formalin before being dehydrated in graded ethanol baths, cleared in xylene and embedded in paraffin. Histological sectioning began at the centre of the graft to avoid the effects of the cuff. 3 µm-thick cross-sections were made throughout the dissected tissue. Immunohistochemical analysis was performed using antibodies to aggrecan (1:100; Abcam, ab36861), the DPEAAE neoepitope of versican (1:100; Abcam, ab19345) and decorin (1:100; Sigma-Aldrich, SAB2100539). Negative controls were generated with 10% solution of normal goat serum (Vector labs). Slides were incubated with the primary antibody overnight at 4 °C. After washing, a secondary antibody (1:400, biotin conjugated goat anti-rabbit IgG; Vector labs) was applied for 30 min at room temperature. Specific immunohistochemical staining was detected by thoroughly washing slides and subsequently incubating with horseradish peroxidase (HRP) labelled avidin D (Vector labs) for 2 h at room temperature. The final detection step was carried out using a DAB (diaminobenzidine) peroxidase kit according to the manufactures instructions (Vector Labs). Sections were counterstained with haematoxylin and mounted. Images were taken by a Leica DM 2000 microscope interfaced to LAS software (version 4.3.0; Leica microsystems).

***Adamts5* Δcat Mice.** Animal experiments were approved by the U.K. authorities (licensed to Q. Xu, PPL70/7266). 10 to 12-week-old wild-type mice (WT) or mice lacking the catalytic domain of Adamts5 (*Adamts5* Δcat)[8] were utilized for the following experiments. For each of the experiments between 5 - 7 mice per group were utilized. Aortas were dissected from WT or *Adamts5* Δcat mice from the aortic root to the iliac bifurcation. Periaortic fat and lymph nodes were removed under a dissecting microscope. Cleaned aortas were washed, diced and subjected to a three-step extraction method in order to enrich the ECM proteins as

described under ‘ECM extraction’. 15 µg of deglycosylated proteins of the GuHCl fraction were subjected to in-solution digestion and analysed by LC-MS/MS as described in the manuscript under ‘proteomics analysis in porcine tissue’. The raw files were searched against the UniProt mouse database (UniProtKB/Swiss-Prot Release 2016_02, 16765 protein entries).

Murine Ultrasound Measurements. Animals were placed in the induction chamber and anaesthetised by using 5% isoflurane mixed with 1l/min of 100% oxygen for 45 sec - 1 min. Next, mice were placed in a supine position on the heating pad to maintain body temperature with embedded ECG. 1-1.5% isoflurane mixed with 1 L/min 100% oxygen was subsequently used to maintain a steady state of sedation levels during the entire procedure. A rectal probe was gently inserted to continuously monitor the body temperature. Two-dimensional (2D) echocardiographic images of cardiovascular anatomy were obtained by a single operator. Standard and modified parasternal long axis (PLAX), suprasternal (SS), longitudinal and transverse abdominal (LA, TA) views were obtained using Visual Sonics Vevo 2100. Aortic root dimensions (aortic annulus, sinuses of Valsalva, sinotubular junction) were measured in PLAX. Ascending aortic dimensions were measured in SS where possible, and modified PLAX if SS views were considered inadequate, in systole and diastole. Abdominal aorta measurements were made in the TA view. All measurements were performed offline on (Vevo software version 1.7) by two independent investigators.

Murine Cardiovascular Magnetic Resonance Imaging (CMR). CMR imaging was performed on a 7T horizontal MR scanner (Varian, Palo Alto, CA) with mice positioned in the prone position. The gradient coil had an inner diameter of 12 cm, 1,000 mT/m (100G/cm) gradient strength, and rise-time of 120 µs. A quadrature transmit/receive coil (RAPID Biomedical). Anesthesia was maintained with 1.5% isoflurane/98.5% oxygen, and body temperature was maintained at 37 °C using a warm air fan (SA Instruments, Stony Brook, NY). The ECG was monitored via 2 metallic needles placed subcutaneously into the front paws. A pressure-transducer for respiratory gating was placed on the abdomen. To synchronize data acquisition with the ECG and to compensate for respiratory motion, simultaneous ECG triggering and respiration gating (SA Instruments) were applied. Late

gadolinium enhancement MRI was performed 20 min after intraperitoneal injection of a 30 μl bolus of 0.5 mmol/kg gadolinium-diethylenetri-amine-pentaacetic acid (Magnevist, Schering Healthcare). Cine-MRI was used to acquire gadolinium enhanced images. Ejection fraction was obtained from cine-MR images according to a previously published method.[9]

Murine Blood Pressure Measurements. Blood pressure was measured directly via implantable radio telemetry device for collection of continuous blood pressure data. Around 200 values were acquired every 5 minutes for 18/20 hours. For each mouse the average value of these 200 points was considered.

Supplemental Table I. Sample characteristics.

Media									
Lifetime [days]	Stent/Balloon	Coronary artery	Sex	Weight [kg]	Age [months]	Size [mm]	AVD [mm]	Target overstretch [mm]	Inflation [atm]
1	BMS	LAD	M	35	3	3.5x15	3.4	4.1	22
		LCX dis	M	35	3	3.5x15	3.0	3.5	12
		RCA prox	F	38	3	3.5x15	3.0	3.6	12
	DES	LAD	F	38	3	3.0x15	3.0	3.5	23
		LCX prox	F	38	3	3.5x15	3.1	3.7	12
		RCA	M	35	3	3.0x15	3.0	3.6	24
	BA early	LCX prox	M	35	3	3.5x15	3.0	3.5	28
		RCA dis	F	38	3	3.5x15	2.9	3.5	12
3	BMS	LAD	M	45	4	3.0x15	2.7	3.2	15
		LCX	F	45	4	3.0x15	2.8	3.3	16
		RCA dis	M	45	4	3.0x15	2.7	3.2	14
	DES	LAD	F	45	4	3.0x15	2.8	3.3	14
		LCX	M	45	4	3.0x15	2.6	3.1	11
		RCA prox	F	45	4	3.0x15	2.7	3.0	14
	BA early	RCA prox	M	45	4	3.0x15	2.8	3.4	16
		RCA dis	F	45	4	3.0x15	2.5	3.0	14
7	BMS	LAD prox	M	32	3	3.5x15	3.1	3.7	14
		LCX prox	F	33.5	3	3.5x15	2.9	3.4	10
		RCA prox	F	33.5	3	3.0x15	2.8	3.4	18
	DES	LAD prox	F	33.5	3	3.5x15	3.0	3.5	9
		LCX prox	M	32	3	3.5x15	3.0	3.6	9
		RCA prox	M	32	3	3.0x15	2.4	2.9	10
14	BA late	LCX prox	M	33	3	3.0x15	2.5	2.9	14
28	BMS	LAD prox	F	29	3	3.5x15	2.9	3.5	10
		LCX prox	F	28	3	3.0x15	2.5	3.0	10
		RCA prox	F	28	3	3.0x15	2.0	2.4	10
	DES	LCX prox	F	29	3	3.5x15	3.0	3.5	9
		RCA medial	F	29	3	3.5x15	3.1	3.5	9
		RCA dis	F	28	3	3.0x15	2.0	2.4	9
	BA late	LAD	F	28	3	3.5x15	2.9	3.5	10
		RCA prox	F	29	3	3.5x15	2.9	3.5	12

Neointima									
Lifetime [days]	Stent-type	Coronary artery	Sex	Weight [kg]	Age [months]	Size [mm]	AVD [mm]	Target overstretch [mm]	Inflation [atm]
28	BMS	LAD prox	F	29	3	3.5x15	2.9	3.5	10
		LCX prox	F	28	3	3.0x15	2.5	3.0	10
		RCA prox	F	28	3	3.0x15	2.0	2.4	10
		RCA prox	M	47	3.5	3.5x8	3.0	3.6	12
		RCA dis	M	47	3.5	3.0x12	2.8	3.4	20
		RCA prox	M	48	3.5	4.0x14	3.4	4.1	12
		LCX	M	48	3.5	3.0x15	2.8	3.4	16
	DES	LCX prox	F	29	3	3.5x15	3.0	3.5	9
		RCA medial	F	29	3	3.5x15	3.1	3.5	9
		RCA dis	F	28	3	3.0x15	2.0	2.4	9
		LAD dis	M	47	3.5	3.0x15	2.6	3.1	12
		LCX med	M	47	3.5	3.5x18	3.2	3.9	18
		RCA dis	M	48	3.5	3.5x15	2.9	3.8	12
		LAD med	M	48	3.5	3.0x18	2.4	2.9	9→12

Clinical characteristics of pigs (lifetime, sex, weight, age at intervention) and PCI specific parameters are shown, including used stent type, balloon/stent size at implantation, average vessel diameter (AVD) as measured by angiography before dilatation, final size of stent/balloon after expansion (target overstretch) as well as the applied inflation pressure. Macroscopically neointima formation was detected in all arteries at 28 days, independent of stent type. Prox = proximal, dis = distal.

Supplemental Table II. Extracellular proteins identified by proteomics analysis in the neointima of stented porcine coronary arteries.

Identified Proteins	UniProt ID	MW (kDa)	BMS Av±SD	DES Av±SD	FC DES/BMS	*P*	FDR
Adipocyte enhancer-binding protein 1	F1SSF7_PIG	128	165.6±93.4	142.4±32.5	0.9	0.554	0.725
Aggrecan	F1SKR0_PIG	238	41.2±69.5	70.8±34.1	1.7	0.338	0.559
Agrin	I3LGD9_PIG	216	116.6±65.1	128.5±40.2	1.1	0.688	0.799
Alpha-1-antitrypsin*	A1AT_PIG	47	33.6±30.2	11.8±8.0	0.4	0.109	0.305
Alpha-2-HS-glycoprotein	FETUA_PIG	38	472.0±223.6	263.1±75.3	0.6	**0.050**	0.203
Annexin A1	ANXA1_PIG	39	231.1±142.4	129.4±45.3	0.6	0.113	0.305
Annexin A2	ANXA2_PIG	39	571.0±247.8	430.9±65.4	0.8	0.192	0.391
Antithrombin-III	Q7M364_PIG	49	92.2±38.7	71.2±18.1	0.8	0.228	0.433
Apolipoprotein A-I	APOA1_PIG	30	619.6±234.8	549.4±270.4	0.9	0.614	0.775
Apolipoprotein A-IV	APOA4_PIG	43	7.6±4.0	10.9±8.3	1.4	0.362	0.581
Apolipoprotein B*	Q29021_PIG	300	27.7±33.0	11.8±15.5	0.4	0.278	0.480
Apolipoprotein C-III	APOC3_PIG	11	16.2±9.2	18.8±11.9	1.2	0.658	0.799
Apolipoprotein E	APOE_PIG	37	76.6±73.2	63.8±70.2	0.8	0.745	0.840
Apolipoprotein H	I3LGN5_PIG	29	141.2±84.9	128.0±131.6	0.9	0.827	0.881
Apolipoprotein R	APOR_PIG	23	27.0±17.6	40.8±22.6	1.5	0.228	0.433
Asporin	F1SUE4_PIG	42	137.6±70.1	80.2±33.0	0.6	0.083	0.257
**Biglycan**	K7GP55_PIG	41	1767.1±214.9	1130.6±169.0	0.6	**0.000**	**0.007**
Bone morphogenic protein 1*	F1RMB2_PIG	114	0.0±0.0	4.9±5.6	n/a	n/a	n/a
Carboxypeptidase-like protein X2	F1SEC6_PIG	86	24.9±26.1	15.8±5.3	0.6	0.397	0.592
Cathepsin D	Q4U1U3_PIG	44	79.6±46.1	34.5±24.3	0.4	**0.047**	0.203
Cathepsin G	F1SGS1_PIG	29	0.4±1.0	0.0±0.0	0.0	n/a	n/a
Cathepsin Z*	A5GFX7_PIG	34	2.9±5.6	0.0±0.0	0.0	n/a	n/a
Chitinase-3-like protein 1	CH3L1_PIG	43	31.8±39.8	2.6±4.6	0.1	n/a	n/a
Chondroadherin*	CHAD_HUMAN	40	0.8±1.3	35.6±25.3	46.5	n/a	n/a
**Clusterin**	CLUS_PIG	52	149.6±28.5	301.7±99.0	2.0	**0.006**	**0.083**
Coatomer subunit alpha*	F1RJX8_PIG	138	16.3±14.7	14.6±7.2	0.9	0.796	0.872
Collagen alpha-1 (I)	CO1A1_PIG	100	2798.6±873.9	1842.9±504.6	0.7	**0.032**	0.182
**Collagen alpha-1 (III)**	F1RYI8_PIG	139	698.6±329.3	224.0±93.9	0.3	**0.008**	**0.092**
Collagen alpha-1 (IV)	M3V819_PIG	160	49.1±20.8	47.8±10.7	1.0	0.887	0.920
Collagen alpha-1 (V)	F1S021_PIG	182	205.5±64.2	137.6±35.9	0.7	**0.036**	0.188
Collagen alpha-1 (VI)	CO6A1_PIG	43	36.4±27.4	19.2±7.1	0.5	0.155	0.342
Collagen alpha-1 (VIII)	F1SKX7_PIG	73	14.1±5.2	19.8±5.7	1.4	0.075	0.252
Collagen alpha-1 (XI)	F1S571_PIG	159	19.4±20.4	10.9±12.2	0.6	0.369	0.581
**Collagen alpha-1 (XII)**	COCA1_PIG	229	484.1±151.0	218.6±50.9	0.5	**0.003**	**0.063**
**Collagen alpha-1 (XV)**	COFA1_PIG	27	68.8±36.2	137.6±20.6	2.0	**0.002**	**0.060**
Collagen alpha-1 (XVIII)	COIA1_PIG	62	216.1±61.8	248.4±36.7	1.1	0.263	0.468
Collagen alpha-1 (XX)	COKA1_PIG	192	1190.7±613.9	440.1±145.6	0.4	**0.017**	0.139
Collagen alpha-2 (I)	F1SFA7_PIG	129	1305.6±466.3	845.6±261.8	0.6	**0.048**	0.203
Collagen alpha-2 (IV)	F1RLL9_PIG	161	89.9±26.6	82.8±13.2	0.9	0.542	0.721
Collagen alpha-2 (V)	Q59IP2_PIG	145	134.8±55.5	66.7±26.4	0.5	**0.018**	0.139
Collagen alpha-2 (VI)	I3LQ84_PIG	106	124.7±110.6	57.2±29.7	0.5	0.163	0.350
Collagen alpha-3 (V)	Q59IP1_PIG	172	2.1±5.5	0.0±0.0	0.0	n/a	n/a
Collagen alpha-3 (VI)	I3LUR7_PIG	342	371.3±357.3	92.9±63.2	0.3	0.086	0.258
Collagen alpha-6 (VI)	CO6A6_PIG	250	8.1±12.9	0.0±0.0	0.0	n/a	n/a
Complement C3	CO3_PIG	187	805.6±730.5	687.7±404.2	0.9	0.717	0.818
Complement component C8B*	A0SEH2_PIG	69	23.1±18.9	27.3±18.4	1.2	0.682	0.799
Complement component C9	A0SEG9_PIG	62	2.4±1.5	6.9±6.4	2.9	0.115	0.305
Connective tissue growth factor	CTGF_PIG	38	1.2±3.1	6.3±7.4	5.4	n/a	n/a
Decorin	PGS2_PIG	40	185.4±59.1	104.5±81.3	0.6	0.057	0.208
Dermatopontin	DERM_PIG	22	55.8±23.6	38.1±14.2	0.7	0.121	0.314
Dystroglycan	I3LD20_PIG	95	3.3±4.4	1.7±2.5	0.5	n/a	n/a
EMILIN-1	F1SDQ5_PIG	107	64.1±34.1	62.4±12.4	1.0	0.905	0.930

EMILIN-2*	F1SBC8_PIG	106	0.5±1.2	0.0±0.0	0.0	n/a	n/a
Fibrillin-1	FBN1_PIG	313	23.8±28.1	10.4±10.8	0.4	0.274	0.480
Fibrinogen beta chain	F1RX37_PIG	56	5135.7±4558.0	4487.1±2656.8	0.9	0.752	0.840
Fibrinogen gamma chain	F1RX35_PIG	50	4287.0±3582.2	3875.7±2485.5	0.9	0.808	0.872
**Fibromodulin**	F1S6B5_PIG	44	549.7±94.2	350.4±115.8	0.6	**0.004**	**0.071**
Fibronectin	F1SS24_PIG	272	10317.1±3240.8	11617.1±2173.3	1.1	0.398	0.592
Fibronectin type III domain-containing protein 1	F1SB59_PIG	204	26.8±41.2	11.7±9.9	0.4	n/a	n/a
Fibulin-1	F1SM61_PIG	78	120.4±47.0	149.3±43.4	1.2	0.255	0.465
Fibulin-2	FBLN2_PIG	54	20.9±22.8	16.5±14.2	0.8	0.670	0.799
Fibulin-3	F8SIP2_PIG	54	190.2±94.1	155.0±53.8	0.8	0.412	0.602
Fibulin-4*	F1RU22_PIG	49	36.2±11.8	38.3±7.7	1.1	0.694	0.799
Fibulin-5	F1SD87_PIG	50	300.0±137.6	351.1±84.6	1.2	0.422	0.608
Filamin-C*	F1SMN5_PIG	290	310.9±151.2	387.0±102.9	1.2	0.295	0.502
Galectin-1	LEG1_PIG	15	353.4±61.5	265.0±52.4	0.7	**0.014**	0.131
Galectin-3	A3EX84_PIG	27	87.2±91.7	29.8±14.2	0.3	0.151	0.342
Galectin-3-binding protein	M3V7X9_PIG	61	14.3±11.8	52.4±42.3	3.7	0.056	0.208
Galectin-9*	F1RJ33_PIG	40	1.3±1.7	0.3±0.6	0.3	n/a	n/a
Gelatinase A*	Q95JA4_PIG	74	6.6±8.5	0.5±0.9	0.1	n/a	n/a
Gelsolin	GELS_PIG	85	1242.1±295.1	1123.4±194.5	0.9	0.394	0.592
Hemicentin-1*	HMCN1_PIG	180	1.3±2.2	0.7±1.8	0.5	n/a	n/a
Hyaluronan and proteoglycan link protein 1	HPLN1_PIG	40	7.7±10.8	16.2±15.4	2.1	0.257	0.465
Insulin-like growth factor-binding protein 7	C7EDN1_PIG	29	395.2±201.4	468.1±122.0	1.2	0.432	0.615
Inter-alpha-trypsin inhibitor heavy chain H1*	ITIH1_PIG	100	145.1±77.4	143.1±46.0	1.0	0.955	0.964
Inter-alpha-trypsin inhibitor heavy chain H2	ITIH2_PIG	105	95.1±63.8	63.5±16.7	0.7	0.247	0.462
Intercellular adhesion molecule 1*	F1S3J9_PIG	58	0.8±2.2	0.0±0.0	0.0	n/a	n/a
Kininogen-1*	KNG1_PIG	48	20.6±15.8	14.8±11.8	0.7	0.450	0.625
**Lactadherin**	MFGM_PIG	46	253.9±104.7	407.3±57.5	1.6	**0.008**	**0.092**
Laminin subunit alpha-4	F1RZM4_PIG	164	75.8±28.8	74.4±31.8	1.0	0.933	0.950
Laminin subunit beta-1	F1SAE9_PIG	199	246.4±69.2	265.6±65.1	1.1	0.604	0.773
Laminin subunit beta-2	F1SPT5_PIG	179	39.4±46.5	39.7±31.1	1.0	0.987	0.987
Laminin subunit gamma-1	F1S663_PIG	177	406.9±140.2	440.3±100.8	1.1	0.619	0.775
Latent TGFβ-binding protein 1	F1S405_PIG	148	28.3±9.3	19.5±6.9	0.7	0.068	0.242
Latent TGFβ-binding protein 2	F1S2T5_PIG	196	174.6±79.5	198.0±58.4	1.1	0.544	0.721
Latent TGFβ-binding protein 4	LTBP4_PIG	112	38.5±26.7	35.6±14.9	0.9	0.811	0.872
Leukocyte elastase inhibitor	ILEU_PIG	43	27.5±31.0	7.0±5.3	0.3	0.134	0.339
**Lumican**	F1SQ09_PIG	39	490.7±128.7	145.1±44.0	0.3	**0.000**	**0.012**
Lysyl oxidase homolog 1	F1SIC9_PIG	65	68.5±29.0	63.0±19.0	0.9	0.687	0.799
Macrophage capping protein	F1SVB0_PIG	39	109.4±87.6	62.1±38.0	0.6	0.226	0.433
Matrilin-4*	F1SDQ7_PIG	69	0.5±1.2	0.7±1.9	1.5	n/a	n/a
Matrix Gla protein	MGP_PIG	12	27.1±5.8	61.9±31.1	2.3	**0.025**	0.163
Matrix-remodeling-associated protein 5	F1RZ07_PIG	308	47.9±48.7	13.7±8.9	0.3	0.114	0.305
Metalloproteinase inhibitor 1*	TIMP1_PIG	23	0.4±0.9	0.0±0.0	0.0	n/a	n/a
Mimecan	I3L9T6_PIG	25	108.2±29.8	96.8±26.8	0.9	0.467	0.642
Myeloperoxidase	K7GRV6_PIG	84	4.5±8.1	0.0±0.0	0.0	n/a	n/a
Nidogen-1	NID1_PIG	50	29.1±17.4	76.6±35.8	2.6	**0.012**	0.127
Nidogen-2	F1SFF3_PIG	152	948.4±283.4	713.1±127.3	0.8	0.079	0.256
Osteoclast-stimulating factor 1*	OSTF1_PIG	24	13.8±11.9	6.3±5.4	0.5	0.166	0.350
Papilin	F1S3J7_PIG	141	0.1±0.2	0.7±1.8	9.3	n/a	n/a
Peptidyl-prolyl cis-trans isomerase A	PPIA_PIG	18	732.6±221.5	646.6±224.5	0.9	0.484	0.657

Periostin	F1RS37_PIG	93	2380.0±652.6	1489.0±573.9	0.6	**0.019**	0.139
Perlecan	PGBM_PIG	365	3168.6±652.1	3467.1±535.4	1.1	0.368	0.581
**Peroxidasin homolog**	I3LDA4_PIG	164	13.3±8.3	31.7±10.6	2.4	**0.004**	**0.071**
Pigment epithelium-derived factor	Q0PM28_PIG	46	264.7±46.4	314.9±70.6	1.2	0.146	0.342
Plasma glutamate carboxypeptidase*	PGCP_PIG	52	2.3±3.2	1.3±1.7	0.6	n/a	n/a
Plasminogen	PLMN_PIG	91	272.3±106.8	514.1±199.6	1.9	**0.019**	0.139
Podocan	I3LEB7_PIG	72	60.1±38.7	23.4±10.0	0.4	**0.047**	0.203
Procollagen C-endopeptidase enhancer 1	I3LEE6_PIG	50	40.2±10.6	41.9±11.6	1.0	0.778	0.861
Prolargin	F1S6B4_PIG	43	451.1±102.9	351.1±138.2	0.8	0.153	0.342
Prolow-density lipoprotein receptor-related protein 1	K9IVL7_PIG	505	30.4±26.2	20.5±7.4	0.7	0.372	0.581
Properdin	K7GQR1_PIG	51	57.9±29.3	117.1±61.8	2.0	**0.049**	0.203
Prophenin and tritrpticin precursor	PF11_PIG	24	230.0±297.6	7.5±8.9	0.0	0.095	0.279
Proteoglycan 4	I3L5Z3_PIG	149	0.9±1.5	0.0±0.0	0.0	n/a	n/a
Reticulon-3*	RTN3_PIG	105	0.0±0.0	0.6±1.5	n/a	n/a	n/a
RPE-spondin	RPESP_PIG	32	1.1±3.0	1.9±3.3	1.6	n/a	n/a
Secreted frizzled-related protein 1	I3LB66_PIG	35	12.1±6.0	9.2±6.4	0.8	0.400	0.592
Secreted frizzled-related protein 3*	F1RYL4_PIG	36	3.3±3.6	2.5±1.1	0.8	0.591	0.766
Secreted phosphoprotein 24*	SPP24_PIG	23	0.0±0.0	12.4±9.1	n/a	n/a	n/a
Serine protease HTRA1	F1SEH4_PIG	56	138.2±102.9	348.3±183.4	2.5	**0.026**	0.163
Serotransferrin	TRFE_PIG	77	3167.4±2227.2	1765.1±729.6	0.6	0.156	0.342
Serum amyloid P-component	SAMP_PIG	26	3.2±6.3	14.2±14.9	4.4	n/a	n/a
SPARC	SPRC_PIG	34	128.6±37.6	154.1±75.8	1.2	0.448	0.625
Spondin-1	SPON1_PIG	66	5.7±4.8	0.7±1.0	0.1	**0.033**	0.182
Sulfhydryl oxidase	F1S682_PIG	81	19.2±11.1	11.8±6.2	0.6	0.154	0.342
Superoxide dismutase [Cu-Zn]	Q007T6_PIG	26	236.2±133.9	155.7±49.9	0.7	0.176	0.364
T-cadherin*	A8D737_PIG	78	22.0±12.0	13.9±6.9	0.6	0.153	0.342
Tenascin	TENA_PIG	191	861.4±309.9	1192.9±318.3	1.4	0.072	0.248
Tetranectin	F1SRC8_PIG	22	156.6±34.1	171.5±75.3	1.1	0.644	0.798
TGFβ-induced protein ig-h3	BGH3_PIG	74	64.8±20.5	55.1±13.0	0.9	0.317	0.532
Thrombospondin-1	K7GPJ3_PIG	120	344.4±124.1	565.3±195.2	1.6	**0.030**	0.178
Thrombospondin-4*	F1RF28_PIG	104	8.9±7.9	33.3±26.4	3.7	0.051	0.203
Translationally-controlled tumor protein*	TCTP_PIG	20	50.9±23.5	49.1±19.5	1.0	0.874	0.914
Tryptase	TRYT_PIG	30	0.1±0.3	0.0±0.0	0.0	n/a	n/a
Tubulointerstitial nephritis antigen-like	F1SVA2_PIG	52	98.8±33.3	128.6±24.4	1.3	0.083	0.257
Versican	F1REZ2_PIG	369	1073.4±242.9	797.6±234.6	0.7	0.052	0.203
Vitamin D-binding protein	I3LN42_PIG	53	143.0±62.4	106.4±38.1	0.7	0.215	0.429
**Vitronectin**	VTNC_PIG	53	419.9±72.3	1038.1±338.7	2.5	**0.003**	**0.063**
von Willebrand factor A domain-containing protein 1	F1RJE3_PIG	44	8.3±6.4	10.0±7.6	1.2	0.660	0.799
WD repeat-containing protein 1	K9IVR7_PIG	66	288.7±36.1	297.6±112.3	1.0	0.848	0.895

P values for differential expression between neointimal DES and BMS are based on unpaired Student's *t*-tests with unequal variance (n=7 [BMS], n=7 [DES]). *T*-test was not performed if a protein was undetectable in the majority of samples from 1 of the 2 groups compared. Results in bold indicate $P < 0.05$. Values are average (Av) total ion current (TIC) x 10^6 ± standard deviation (SD). n/a denotes not applicable, FC denotes fold change. FDR denotes false discovery rate. The FDR threshold was set at 10%. Protein changes with $P < 0.05$ and a FDR of <10% are highlighted in bold. Proteins only identified in the neointima, but not media are marked with*.

Supplemental Table III. Extracellular proteins identified by proteomics in the media of stented porcine coronary arteries.

		**Bare-metal stent** Total ion current x 10^6 Av±SD						**Drug-eluting stent** Total ion current x 10^6 Av±SD					
**Identified Proteins**	**UniProt ID**	**BMS 1**	**BMS 3**	**BMS 7**	**BMS 28**	***P*** **BMS**	**FDR**	**DES 1**	**DES 3**	**DES 7**	**DES 28**	***P*** **DES**	**FDR**
Adipocyte enhancer-binding protein 1	F1SSF7_PIG	0.3±0.5	0.2±0.3	1.6±1.3	1.1±0.5	0.136	0.326	0.1±0.2	0.0±0.0	1.0±0.9	6.2±4.8	**0.046**	0.140
**Aggrecan**	F1SKR0_PIG	6.5±2.3	2.7±0.9	18.0±11.4	28.2±9.0	**0.010**	**0.088**	5.2±2.1	10.9±9.3	18.3±7.6	52.5±11.1	**0.000**	**0.011**
Agrin	I3LGD9_PIG	5.7±2.5	5.4±2.8	8.7±1.1	9.4±1.7	0.112	0.310	5.7±3.3	5.2±3.5	8.8±1.3	6.2±1.6	0.400	0.523
Alpha-2-HS-glycoprotein	FETUA_PIG	1.4±1.4	1.6±1.5	2.6±1.9	5.2±8.5	0.717	0.751	4.0±2.1	3.7±2.4	3.7±3.7	7.8±7.5	0.623	0.679
Annexin A1	ANXA1_PIG	1.8±0.8	2.3±1.0	1.7±0.6	2.0±2.2	0.943	0.943	2.4±1.1	3.7±1.6	3.7±2.9	3.7±0.9	0.765	0.793
**Annexin A2**	ANXA2_PIG	10.8±1.8	14.2±4.7	24.3±4.3	37.2±31.9	0.265	0.431	9.5±1.5	12.7±7.0	20.8±4.3	28.1±7.7	**0.016**	**0.066**
Antithrombin-III	Q7M364_PIG	2.5±1.3	0.8±0.5	0.0±0.0	0.7±0.8	**0.028**	0.161	1.4±0.7	14.2±17.7	2.1±1.1	1.7±0.7	0.289	0.432
Apolipoprotein A-I	APOA1_PIG	18.7±4.3	11.0±8.0	21.2±12.5	25.6±16.3	0.484	0.580	17.5±2.2	26.8±13.5	19.1±13.2	24.8±10.0	0.686	0.732
Apolipoprotein A-IV	APOA4_PIG	0.1±0.1	0.0±0.0	0.1±0.1	0.4±0.6	0.394	0.536	0.0±0.1	1.7±1.1	0.4±0.5	0.5±0.3	0.053	0.142
**Apolipoprotein C-III**	APOC3_PIG	4.4±2.5	2.3±1.4	0.6±0.6	0.7±0.7	**0.049**	0.231	4.3±1.7	5.6±2.9	0.5±0.6	0.8±0.4	**0.016**	**0.066**
**Apolipoprotein E**	APOE_PIG	15.7±4.0	11.3±7.0	1.8±1.7	1.6±0.7	**0.006**	**0.077**	8.9±4.2	82.2±70.2	8.9±6.2	1.9±2.4	0.071	0.156
Apolipoprotein H	I3LGN5_PIG	21.7±6.2	20.3±5.4	18.7±2.7	29.4±18.3	0.603	0.657	26.7±5.1	36.6±27.2	29.3±14.7	59.5±10.6	0.139	0.260
**Apolipoprotein R**	APOR_PIG	1.6±1.1	1.0±0.3	0.8±0.8	0.3±0.3	0.213	0.395	2.6±1.6	6.8±2.7	0.9±0.8	0.5±0.5	**0.006**	**0.034**
**Asporin**	F1SUE4_PIG	78.2±16.2	62.0±11.9	86.2±17.1	150.3±37.5	**0.007**	**0.077**	77.5±16.5	81.7±37.1	90.1±30.2	157.3±43.8	0.059	0.142
**Biglycan**	K7GP55_PIG	796.0±130.8	648.1±186.6	952.7±42.3	1503.4±443.4	**0.014**	0.104	748.6±200.3	750.0±367.0	1002.9±236.7	1972.6±202.2	**0.001**	**0.011**
Carboxypeptidase-like protein X2	F1SEC6_PIG	0.0±0.0	0.0±0.0	0.0±0.0	0.0±0.0	n/a	n/a	0.0±0.0	0.0±0.0	0.0±0.0	0.4±0.2	n/a	n/a
Cathepsin D	Q4U1U3_PIG	1.6±0.5	1.9±0.3	7.2±4.8	8.8±13.1	0.523	0.604	1.1±1.5	2.3±1.6	4.1±3.6	3.7±1.8	0.412	0.523
Cathepsin G	F1SGS1_PIG	0.5±0.3	0.0±0.0	0.0±0.0	0.0±0.0	n/a	n/a	0.8±0.8	0.8±0.7	0.1±0.1	0.0±0.0	0.201	0.341
Chitinase-3-like protein 1	CH3L1_PIG	0.0±0.0	0.0±0.0	0.0±0.0	0.3±0.5	n/a	n/a	0.0±0.0	0.0±0.0	0.0±0.0	0.0±0.0	n/a	n/a
Chondroitin sulfate proteoglycan 4*	CSPG4_HUMAN	0.1±0.1	0.0±0.0	0.3±0.5	0.0±0.0	0.415	0.546	0.0±0.0	0.0±0.0	0.0±0.0	0.0±0.0	n/a	n/a
Clusterin	CLUS_PIG	98.2±51.7	59.8±17.0	45.6±16.8	16.4±10.7	**0.048**	0.231	59.0±19.6	273.4±202.6	61.0±24.0	21.2±10.8	0.060	0.142
Collagen alpha-1 (I)	CO1A1_PIG	2915.1±375.6	2913.2±993.1	4118.5±199.0	2622.2±1399.4	0.244	0.419	2749.1±797.9	1973.1±1213.2	3353.0±487.7	3996.0±273.1	0.062	0.142
Collagen alpha-1 (II)*	I3LSV6_PIG	108.4±22.4	127.9±54.4	149.6±3.8	68.4±34.1	0.090	0.276	100.9±39.9	77.0±38.5	122.7±62.7	116.6±15.0	0.584	0.669
**Collagen alpha-1 (III)**	F1RYI8_PIG	332.8±111.3	571.8±520.0	454.4±127.6	472.3±201.4	0.797	0.807	302.2±59.7	185.0±119.2	318.7±83.5	480.3±67.1	**0.019**	**0.072**
Collagen alpha-1 (IV)	M3V819_PIG	3.1±2.1	5.7±1.9	7.5±2.2	8.8±3.3	0.091	0.276	2.5±0.4	4.2±2.5	6.6±0.9	4.3±1.2	0.055	0.142
**Collagen alpha-1 (V)**	F1S021_PIG	81.8±19.0	108.4±42.0	181.5±36.4	174.3±88.5	0.128	0.325	82.2±11.7	109.9±66.9	126.0±20.7	251.3±25.0	**0.003**	**0.019**
Collagen alpha-1 (VI)	CO6A1_PIG	4.5±1.2	3.6±3.9	2.0±0.4	10.3±8.4	0.221	0.395	4.8±2.2	6.2±5.1	2.6±2.9	1.8±0.3	0.371	0.507
**Collagen alpha-1 (VIII)**	F1SKX7_PIG	0.2±0.3	0.2±0.4	1.5±0.6	0.5±0.4	**0.013**	0.104	0.1±0.2	0.4±0.4	1.8±0.9	3.2±1.0	**0.002**	**0.019**
**Collagen alpha-1 (XI)**	F1S571_PIG	48.9±9.7	50.3±22.3	111.5±27.7	103.7±54.7	0.088	0.276	45.9±2.4	62.4±36.9	60.7±17.8	190.8±17.9	**0.000**	**0.006**
**Collagen alpha-1 (XII)**	COCA1_PIG	7.1±0.5	3.4±2.9	17.2±16.6	46.9±52.0	0.273	0.438	5.7±0.7	3.8±1.8	14.2±10.6	53.7±15.8	**0.001**	**0.011**
**Collagen alpha-1 (XV)**	COFA1_PIG	3.2±1.0	2.5±0.9	9.0±2.7	9.5±4.8	**0.028**	0.161	2.3±1.8	2.1±1.2	10.0±2.2	14.1±3.8	**0.001**	**0.011**
Collagen alpha-1 (XVIII)	COIA1_PIG	10.5±3.5	12.3±7.3	11.4±1.1	6.7±1.3	0.420	0.546	8.5±3.5	12.9±5.7	12.2±4.8	1.6±0.3	**0.035**	0.115
**Collagen alpha-1 (XX)**	COKA1_PIG	14.6±12.5	5.7±4.3	22.3±9.2	60.8±43.8	0.081	0.276	8.2±2.6	4.0±2.6	24.8±9.2	22.2±2.9	**0.003**	**0.019**

**Collagen alpha-2 (I)**	F1SFA7_PIG	2540.2±281.6	2097.8±569.3	3114.0±344.6	2616.7±1337.8	0.482	0.580	2441.6±563.3	1949.6±1173.6	2966.9±215.8	4329.5±613.6	**0.019**	**0.072**
Collagen alpha-2 (IV)	F1RLL9_PIG	3.1±1.8	6.9±2.4	7.2±4.2	7.8±0.2	0.184	0.365	1.9±0.6	4.1±3.7	8.4±3.2	2.1±0.4	**0.038**	0.120
Collagen alpha-2 (V)	Q59IP2_PIG	84.2±45.2	108.2±40.4	172.3±73.6	173.7±84.4	0.281	0.444	87.0±14.8	91.2±57.0	156.5±6.4	200.7±119.1	0.188	0.328
Collagen alpha-2 (VI)	I3LQ84_PIG	13.4±3.9	15.3±15.9	7.1±1.6	41.7±41.5	0.309	0.461	14.8±5.6	23.3±24.5	10.3±4.8	8.5±3.2	0.534	0.630
Collagen alpha-2 (XI)*	A5D9K7_PIG	23.6±4.3	36.2±12.2	49.2±10.5	37.8±22.7	0.250	0.420	22.2±5.9	32.8±16.0	30.3±10.1	52.3±10.3	0.055	0.142
Collagen alpha-3 (V)	Q59IP1_PIG	1.1±0.7	1.6±1.2	1.9±0.8	5.8±5.3	0.220	0.395	1.2±0.7	1.9±2.1	2.1±1.0	3.2±1.3	0.402	0.523
Collagen alpha-3 (VI)	I3LUR7_PIG	42.7±11.3	38.2±40.2	16.2±6.8	92.4±98.5	0.411	0.546	32.6±15.3	41.5±39.4	21.3±16.8	18.4±11.3	0.624	0.679
Collagen alpha-6 (VI)	CO6A6_PIG	0.0±0.0	0.0±0.0	0.0±0.0	14.1±24.5	n/a	n/a	0.0±0.0	0.0±0.0	0.0±0.0	0.0±0.0	n/a	n/a
Complement C3	CO3_PIG	4.3±1.1	0.1±0.2	0.6±0.3	1.8±3.1	0.063	0.249	3.3±1.4	14.2±20.9	1.4±1.6	0.4±0.6	0.405	0.523
Complement component C9	A0SEG9_PIG	0.0±0.0	0.0±0.0	0.0±0.0	0.0±0.0	n/a	n/a	0.0±0.0	1.0±1.7	0.0±0.0	0.0±0.0	n/a	n/a
Connective tissue growth factor	CTGF_PIG	0.4±0.3	0.3±0.5	0.7±0.6	0.0±0.0	0.308	0.461	0.8±0.4	0.4±0.3	0.7±0.4	0.1±0.2	0.212	0.345
**Decorin**	PGS2_PIG	581.1±67.5	418.8±208.1	411.1±91.6	1166.4±602.8	0.064	0.249	610.5±85.3	554.8±357.0	441.8±67.1	1145.6±50.6	**0.008**	**0.039**
**Dermatopontin**	DERM_PIG	52.5±13.7	43.6±33.0	45.1±3.3	126.2±69.7	0.090	0.276	64.5±12.2	59.9±38.4	80.7±23.7	150.5±20.2	**0.008**	**0.039**
Dystroglycan	I3LD20_PIG	1.2±1.1	0.6±0.5	0.7±0.2	0.7±0.6	0.729	0.756	0.9±0.6	0.8±1.1	0.6±0.2	0.8±0.5	0.945	0.953
EMILIN-1	F1SDQ5_PIG	6.1±3.2	8.3±2.1	7.9±2.5	10.7±2.4	0.261	0.431	5.6±1.8	6.4±2.8	9.1±1.0	8.8±3.8	0.316	0.461
Fibrillin-1	FBN1_PIG	0.2±0.4	0.8±1.4	0.0±0.0	0.0±0.0	0.519	0.604	0.2±0.3	0.0±0.0	0.0±0.0	0.0±0.0	n/a	n/a
**Fibrinogen beta chain**	F1RX37_PIG	1240.6±374.6	268.9±219.0	49.0±38.0	3.3±0.7	**0.000**	**0.025**	1240.6±892.3	2814.7±3732.6	77.6±53.1	53.8±46.6	0.317	0.461
**Fibrinogen gamma chain**	F1RX35_PIG	1124.9±400.8	267.0±197.3	43.8±31.0	8.0±0.7	**0.001**	**0.025**	994.9±674.2	2014.6±2519.2	69.7±42.6	39.7±31.3	0.277	0.431
**Fibromodulin**	F1S6B5_PIG	144.8±77.4	73.5±29.1	200.7±36.9	299.2±28.4	**0.002**	**0.046**	135.1±50.9	62.4±47.7	185.0±36.5	598.9±175.3	**0.001**	**0.011**
Fibronectin	F1SS24_PIG	557.8±157.1	983.5±206.3	1109.9±420.0	503.9±198.9	0.055	0.241	527.0±248.9	1237.2±357.0	870.3±360.6	925.0±188.5	0.104	0.205
Fibronectin type III domain-containing protein 1	F1SB59_PIG	0.0±0.0	0.0±0.0	0.0±0.0	0.2±0.2	n/a	n/a	0.0±0.0	0.0±0.0	0.0±0.0	1.0±1.5	n/a	n/a
Fibulin-1	F1SM61_PIG	0.3±0.1	0.0±0.1	0.4±0.3	0.4±0.5	0.438	0.562	0.1±0.2	0.3±0.6	0.4±0.2	2.0±1.5	0.077	0.160
**Fibulin-2**	FBLN2_PIG	0.0±0.0	0.2±0.3	0.3±0.3	0.3±0.4	0.634	0.677	0.0±0.0	0.0±0.0	1.5±1.4	4.0±2.0	**0.011**	**0.052**
Fibulin-3	F8SIP2_PIG	0.0±0.0	0.0±0.0	0.0±0.0	0.2±0.3	n/a	n/a	0.0±0.0	0.0±0.0	0.0±0.0	0.0±0.0	n/a	n/a
Fibulin-5	F1SD87_PIG	11.3±1.8	4.4±1.7	7.6±1.1	17.7±13.6	0.185	0.365	9.5±2.7	7.4±3.1	7.3±3.9	7.8±1.5	0.772	0.793
Galectin-1	LEG1_PIG	16.2±4.0	23.5±12.5	40.8±10.8	30.3±8.2	0.062	0.249	23.7±5.0	14.6±10.3	34.3±3.1	25.7±8.6	0.062	0.142
**Galectin-3**	A3EX84_PIG	0.1±0.1	0.2±0.3	1.0±0.2	3.9±6.0	0.421	0.546	0.2±0.2	0.1±0.1	0.5±0.4	1.1±0.2	**0.002**	**0.017**
Galectin-3-binding protein	M3V7X9_PIG	0.4±0.8	2.4±3.2	4.1±1.5	0.7±0.6	0.127	0.325	0.0±0.0	1.3±2.0	1.8±1.5	1.3±0.9	0.475	0.579
Gelsolin	GELS_PIG	217.8±17.2	129.6±51.0	160.6±18.1	179.7±80.3	0.246	0.419	227.7±68.3	150.1±78.2	194.7±35.8	155.4±18.4	0.345	0.495
Hyaluronan and proteoglycan link protein 1	HPLN1_PIG	2.6±2.2	3.5±2.7	7.1±2.5	3.4±0.7	0.128	0.325	4.4±2.5	9.6±2.5	10.9±6.5	0.9±0.8	**0.036**	0.116
Hyaluronan and proteoglycan link protein 3*	HPLN3_HUMAN	0.2±0.3	0.4±0.8	0.6±0.7	0.0±0.0	0.552	0.613	0.7±0.6	0.6±1.0	1.0±1.1	0.1±0.2	0.604	0.677
Hyaluronan and proteoglycan link protein 4*	HPLN4_HUMAN	0.0±0.0	0.0±0.0	0.2±0.3	0.0±0.0	n/a	n/a	0.0±0.0	0.6±0.6	0.0±0.0	0.0±0.0	n/a	n/a
**Insulin-like growth factor-binding protein 7**	C7EDN1_PIG	2.0±1.2	4.3±3.8	22.3±2.8	11.1±9.9	**0.008**	**0.080**	2.1±2.3	1.5±1.7	21.0±7.4	8.8±0.8	**0.001**	**0.011**
Inter-alpha-trypsin inhibitor heavy chain H2	ITIH2_PIG	8.4±3.7	4.5±2.1	3.6±2.2	4.1±1.6	0.163	0.356	6.8±1.5	6.4±3.9	5.7±1.8	6.1±5.2	0.985	0.985
**Lactadherin**	MFGM_PIG	7.9±4.8	11.7±5.7	17.8±9.7	19.1±15.3	0.499	0.591	4.8±3.8	9.3±7.2	19.7±5.5	6.1±0.6	**0.022**	**0.081**
**Laminin subunit alpha-4**	F1RZM4_PIG	6.2±1.5	2.1±1.5	3.5±1.5	11.2±3.5	**0.004**	**0.065**	6.7±4.6	3.9±2.2	3.6±3.9	3.9±1.5	0.655	0.705
Laminin subunit beta-1	F1SAE9_PIG	2.8±1.3	1.4±1.2	6.2±4.7	9.4±1.0	**0.020**	0.127	1.6±1.9	1.4±1.0	4.6±2.7	2.0±1.2	0.188	0.328

Laminin subunit beta-2	F1SPT5_PIG	8.8±2.3	8.1±0.2	7.1±0.9	8.7±1.3	0.462	0.577	6.2±3.7	11.0±2.8	8.3±5.3	4.3±1.9	0.205	0.343
Laminin subunit gamma-1	F1S663_PIG	41.7±11.2	35.9±1.9	38.7±5.3	47.8±8.8	0.330	0.477	36.6±21.7	39.7±6.9	42.7±18.6	16.7±4.9	0.213	0.345
Latent-TGFß-binding protein 1	F1S405_PIG	0.7±0.7	0.4±0.1	0.3±0.2	0.2±0.2	0.526	0.604	0.3±0.1	0.5±0.5	0.6±0.5	0.0±0.0	0.285	0.432
**Latent-TGFß-binding protein 2**	F1S2T5_PIG	2.5±1.3	2.9±2.0	5.5±1.0	2.7±2.4	0.213	0.395	2.4±1.1	2.4±1.8	10.2±0.8	9.1±1.6	**0.000**	**0.006**
Latent-TGFß-binding protein 4	LTBP4_PIG	3.7±1.9	0.1±0.1	0.0±0.0	4.9±5.6	0.177	0.365	5.5±1.7	1.1±1.7	1.4±0.6	2.8±2.9	0.074	0.156
Leukocyte elastase inhibitor	ILEU_PIG	0.0±0.0	0.0±0.0	0.0±0.0	0.1±0.2	n/a	n/a	1.0±0.4	1.6±2.8	0.0±0.0	0.0±0.0	0.463	0.577
**Lipoprotein lipase***	LIPL_PIG	1.8±0.4	1.2±2.0	0.0±0.0	0.0±0.0	0.150	0.335	1.4±0.6	6.1±2.7	3.3±2.1	0.0±0.0	**0.012**	**0.052**
Lumican	F1SQ09_PIG	288.9±36.6	204.3±53.9	312.6±49.2	431.5±212.7	0.188	0.365	257.0±73.7	277.8±152.9	282.2±61.7	405.1±59.0	0.286	0.432
Lysyl oxidase homolog 1	F1SIC9_PIG	5.7±5.2	0.7±0.6	3.9±1.2	7.8±3.3	0.110	0.310	6.2±7.8	2.0±1.7	6.6±4.9	11.3±1.1	0.195	0.336
Macrophage-capping protein	F1SVB0_PIG	0.0±0.0	0.0±0.0	0.1±0.1	0.6±1.0	n/a	n/a	0.1±0.1	0.0±0.0	0.2±0.2	0.4±0.2	0.060	0.142
Matrix Gla protein	MGP_PIG	1.2±0.4	1.7±0.1	2.5±1.1	1.3±0.8	0.149	0.335	1.3±0.7	1.3±0.7	2.8±0.3	1.4±0.5	**0.035**	0.115
Matrix-remodeling-associated protein 5	F1RZ07_PIG	0.3±0.4	6.3±5.4	6.5±9.2	7.4±9.7	0.631	0.677	0.1±0.2	3.9±1.9	7.3±9.9	11.5±5.5	0.175	0.321
Mimecan	I3L9T6_PIG	426.0±40.0	220.5±139.0	255.7±34.2	495.6±304.8	0.220	0.395	389.9±27.1	292.7±193.6	283.7±7.3	581.3±152.7	0.061	0.142
Myeloperoxidase	K7GRV6_PIG	1.1±1.1	0.3±0.5	0.0±0.0	0.7±1.2	0.479	0.580	2.6±2.9	4.4±7.5	0.0±0.0	0.0±0.0	0.494	0.589
Nidogen-1	NID1_PIG	1.5±0.8	2.2±2.2	4.3±1.7	6.1±2.5	0.070	0.255	2.0±0.7	3.8±2.2	5.3±2.6	8.1±2.7	**0.049**	0.142
Nidogen-2	F1SFF3_PIG	109.6±14.8	137.7±31.0	155.9±5.2	173.2±56.0	0.187	0.365	104.0±32.6	157.8±56.4	161.3±12.5	96.8±25.8	0.109	0.211
Papilin	F1S3J7_PIG	0.6±1.0	0.3±0.6	0.2±0.2	0.1±0.2	0.800	0.807	0.6±0.5	0.7±0.5	0.3±0.3	0.0±0.0	0.218	0.349
Pentraxin-related protein PTX3*	F1SJM0_PIG	0.9±0.8	1.2±1.1	0.0±0.0	0.1±0.1	0.138	0.326	0.8±0.3	0.5±0.9	1.4±1.1	0.0±0.0	0.234	0.369
Peptidyl-prolyl cis-trans isomerase A	PPIA_PIG	2.2±1.0	1.5±0.7	4.3±1.5	6.2±8.1	0.536	0.604	3.8±1.8	3.6±2.5	5.8±4.0	7.3±2.8	0.385	0.519
**Periostin**	F1RS37_PIG	2.5±2.3	22.6±19.5	100.3±69.3	148.3±199.7	0.361	0.505	0.5±0.5	6.8±7.0	86.6±62.7	287.5±33.2	**0.000**	**0.003**
Perlecan	PGBM_PIG	210.9±42.8	188.9±59.9	245.7±21.6	384.9±98.0	**0.019**	0.127	215.7±38.4	237.8±121.6	321.6±78.5	336.2±111.9	0.359	0.506
Peroxidasin homolog	I3LDA4_PIG	0.0±0.0	0.3±0.3	0.2±0.1	0.2±0.4	0.565	0.622	0.0±0.0	0.0±0.0	0.1±0.2	0.0±0.0	n/a	n/a
Pigment epithelium-derived factor	Q0PM28_PIG	0.4±0.5	0.5±0.9	2.2±0.9	0.9±1.2	0.134	0.326	0.3±0.5	0.2±0.3	3.3±2.7	1.9±1.3	0.101	0.203
**Plasminogen**	PLMN_PIG	57.8±54.2	79.7±55.9	10.7±5.6	2.1±1.8	0.114	0.310	34.6±16.9	146.3±54.8	11.2±6.5	2.6±1.8	**0.001**	**0.011**
Podocan	I3LEB7_PIG	0.8±0.4	0.1±0.2	1.1±0.8	2.8±1.6	**0.030**	0.163	0.6±0.7	0.5±0.3	0.9±1.1	1.0±0.8	0.888	0.905
Procollagen C-endopeptidase enchancer 1	I3LEE6_PIG	0.2±0.1	0.1±0.1	0.8±0.5	0.7±0.3	0.069	0.255	0.2±0.2	0.1±0.1	2.7±3.8	1.1±0.5	0.366	0.506
**Prolargin**	F1S6B4_PIG	269.7±21.2	200.2±42.2	300.6±54.5	608.6±187.3	**0.005**	**0.065**	263.7±49.7	255.3±109.7	341.3±134.5	995.5±337.7	**0.004**	**0.024**
Prolow-density lipoprotein receptor-related protein 1	K9IVL7_PIG	1.1±0.3	1.4±1.6	3.5±3.5	4.2±2.1	0.295	0.454	1.4±1.2	0.7±0.5	3.6±2.8	4.5±1.2	0.066	0.147
Properdin	K7GQR1_PIG	0.7±0.6	0.3±0.3	0.0±0.0	0.3±0.3	0.228	0.401	0.6±0.7	0.6±0.7	0.3±0.5	0.2±0.1	0.749	0.783
**Prophenin and tritrpticin precursor**	PF11_PIG	63.9±15.1	16.9±10.5	2.1±1.0	8.0±13.4	**0.001**	**0.025**	94.1±47.6	186.5±155.5	9.8±2.0	1.1±1.6	0.074	0.156
Proteoglycan 4	I3L5Z3_PIG	0.5±0.2	0.0±0.0	0.0±0.0	0.0±0.0	n/a	n/a	0.0±0.1	0.1±0.2	0.0±0.0	0.0±0.0	0.554	0.646
RPE-spondin	RPESP_PIG	4.8±2.1	7.9±7.7	5.8±3.3	1.5±1.3	0.393	0.536	9.0±9.3	4.7±2.9	9.3±5.6	2.4±0.4	0.415	0.523
**Secreted frizzled-related protein 1**	I3LB66_PIG	0.0±0.0	0.0±0.0	0.4±0.6	0.9±0.9	0.183	0.365	0.0±0.0	0.0±0.0	0.2±0.3	0.7±0.0	**0.001**	**0.011**
Serine protease HTRA1	F1SEH4_PIG	7.0±1.7	10.8±4.9	46.7±13.0	45.6±41.4	0.100	0.296	5.5±2.7	6.5±3.8	37.4±17.4	37.2±21.0	**0.029**	0.101
Serotransferrin	TRFE_PIG	0.4±0.4	2.3±0.7	1.7±1.8	3.2±5.6	0.710	0.751	3.0±2.3	6.0±2.7	1.6±2.0	6.6±9.7	0.624	0.679

Serum amyloid P-component	SAMP_PIG	0.1±0.1	0.0±0.0	0.0±0.0	0.0±0.0	n/a	n/a	0.1±0.1	0.0±0.0	0.0±0.0	0.0±0.1	0.713	0.753
**SPARC**	SPRC_PIG	0.3±0.6	3.6±1.3	3.5±1.4	0.8±1.0	**0.010**	**0.088**	0.4±0.7	0.5±0.7	3.2±1.9	4.7±1.2	**0.008**	**0.039**
Spondin-1	SPON1_PIG	0.0±0.0	0.1±0.1	0.5±0.5	0.4±0.7	0.449	0.569	0.1±0.1	0.1±0.2	0.5±0.3	0.9±0.8	0.187	0.328
Sulfhydryl oxidase	F1S682_PIG	0.0±0.0	0.0±0.0	0.0±0.0	0.0±0.0	n/a	n/a	0.0±0.0	0.1±0.2	0.0±0.0	0.0±0.0	n/a	n/a
Superoxide dismutase [Cu-Zn]	Q007T6_PIG	1.1±0.4	0.2±0.2	1.6±0.6	1.3±0.7	**0.042**	0.215	1.8±1.5	0.2±0.2	1.3±0.3	2.1±0.6	0.098	0.200
**Target of Nesh-SH3***	F1SL03_PIG	3.1±1.9	0.9±1.5	1.3±2.0	4.1±4.5	0.466	0.577	4.3±2.0	0.8±0.8	3.3±1.0	13.5±5.4	**0.004**	**0.024**
**Tenascin**	TENA_PIG	64.2±20.0	52.4±13.9	257.6±39.4	168.2±84.9	**0.003**	**0.046**	44.9±12.4	82.6±47.7	168.9±41.0	106.5±33.4	**0.017**	**0.070**
Tenascin XB*	A5A8W4_PIG	5.5±2.3	4.4±7.1	3.4±4.8	9.4±11.0	0.747	0.769	5.7±4.1	5.9±5.4	1.8±1.4	6.1±5.3	0.586	0.669
Tetranectin	F1SRC8_PIG	1.6±0.5	0.9±0.8	2.5±2.0	2.3±0.4	0.324	0.477	1.7±0.8	2.2±2.0	3.5±2.6	6.7±2.1	0.059	0.142
TGFß-1*	TGFB1_PIG	0.0±0.0	0.0±0.0	0.0±0.0	0.0±0.0	n/a	n/a	0.0±0.0	0.2±0.3	0.0±0.0	0.0±0.0	n/a	n/a
TGFß-3*	K7GSJ9_PIG	0.0±0.0	0.0±0.0	0.0±0.0	0.0±0.0	n/a	n/a	0.0±0.0	0.0±0.0	0.0±0.0	0.6±0.9	n/a	n/a
TGFß-induced protein ig-h3	BGH3_PIG	19.5±5.0	15.1±11.9	9.5±4.3	27.0±17.6	0.333	0.477	9.0±3.3	15.7±8.3	22.6±19.7	8.6±4.8	0.415	0.523
Throbospondin-1	K7GPJ3_PIG	5.8±2.3	4.2±1.7	7.2±2.1	2.7±0.9	0.073	0.257	4.3±0.9	6.3±2.1	7.1±1.8	6.1±3.2	0.491	0.589
Tryptase	TRYT_PIG	0.0±0.0	0.0±0.0	0.0±0.0	3.8±6.6	n/a	n/a	0.0±0.0	0.0±0.1	0.0±0.0	0.4±0.7	0.475	0.579
Tubulointerstitial nephritis antigen-like	F1SVA2_PIG	83.0±10.1	56.6±25.4	53.2±6.7	65.1±9.5	0.142	0.329	71.7±13.9	67.8±36.6	69.5±11.8	43.5±5.7	0.364	0.506
**Versican**	F1REZ2_PIG	70.2±5.8	103.0±18.5	153.5±7.9	148.5±26.1	**0.001**	**0.025**	112.2±54.7	81.1±31.6	231.4±38.8	398.1±137.4	**0.004**	**0.025**
Vitamin D-binding protein	I3LN42_PIG	8.7±5.6	1.3±1.0	2.7±1.1	1.4±1.8	0.051	0.231	5.3±1.1	9.0±5.5	2.8±1.9	1.4±1.0	0.062	0.142
**Vitronectin**	VTNC_PIG	298.1±250.1	239.7±90.3	148.2±65.3	87.3±94.7	0.352	0.498	202.6±40.6	694.9±156.0	235.4±154.0	82.8±48.1	**0.001**	**0.011**
von Willebrand factor A domain-containing protein 1	F1RJE3_PIG	17.6±8.1	8.6±3.6	10.6±3.4	14.5±6.4	0.294	0.454	18.7±5.6	11.7±6.0	14.6±4.6	7.3±4.4	0.129	0.245
WD repeat-containing protein 1	K9IVR7_PIG	0.0±0.0	0.1±0.2	0.1±0.1	0.1±0.2	0.537	0.604	0.0±0.0	0.0±0.0	0.0±0.1	0.0±0.1	0.595	0.673

P values for changes in BM or DES at different time-points were identified by one-way ANOVA (BMS: n=3 [BMS1/3/7/28], 1-way ANOVA; DES: n=3 [DES 1/3/7/28], 1-way ANOVA). ANOVA was not applied if a protein was detected at only a single time point. Results in bold indicate $P < 0.05$. Values are average (Av) total ion current (TIC) x 10^6 ± standard deviation (SD). n/a denotes not applicable. FDR denotes false discovery rate. The FDR threshold was set at 10%. Protein changes with P <0.05 and a FDR of <10% are highlighted in bold. Proteins only identified in the media, but not neointima are marked with*.

Supplemental Table IV. Extracellular proteins identified by proteomics in the media of balloon dilated porcine coronary arteries.

		Balloon angioplasty Total ion current x 10^6 Av±SD				
**Identified Proteins**	**UniProt ID**	**BA early**	**BA late**	**FC late/early**	***P* BA**	**FDR**
Adipocyte enhancer-binding protein 1	F1SSF7_PIG	0.9±0.6	2.0±1.2	2.1	0.258	0.519
Aggrecan	F1SKR0_PIG	5.3±3.0	17.3±10.7	3.3	0.187	0.431
Agrin	I3LGD9_PIG	5.6±4.0	9.9±1.1	1.8	0.118	0.403
Alpha-2-HS-glycoprotein	FETUA_PIG	3.7±2.2	1.9±1.4	0.5	0.232	0.492
Annexin A1	ANXA1_PIG	3.1±0.8	0.5±0.2	0.2	**0.005**	0.217
Annexin A2	ANXA2_PIG	14.2±10.5	14.9±2.9	1.1	0.898	0.951
Antithrombin-III	Q7M364_PIG	2.5±1.8	0.3±0.5	0.1	n/a	n/a
Apolipoprotein A-I	APOA1_PIG	19.5±7.7	12.7±5.6	0.7	0.231	0.492
Apolipoprotein A-IV	APOA4_PIG	0.0±0.0	0.1±0.1	n/a	n/a	n/a
Apolipoprotein C-III	APOC3_PIG	6.5±3.9	0.2±0.4	0.0	**0.048**	0.301
Apolipoprotein E	APOE_PIG	26.2±20.3	1.2±0.3	0.0	n/a	n/a
Apolipoprotein H	I3LGN5_PIG	23.1±4.4	19.5±8.5	0.8	0.551	0.720
Apolipoprotein R	APOR_PIG	1.9±2.3	0.0±0.0	0.0	n/a	n/a
Asporin	F1SUE4_PIG	61.0±14.8	87.4±40.2	1.4	0.375	0.565
Biglycan	K7GP55_PIG	659.9±210.4	971.4±110.9	1.5	0.056	0.301
Carboxypeptidase-like protein X2	F1SEC6_PIG	0.0±0.0	0.0±0.0	n/a	n/a	n/a
Cathepsin D	Q4U1U3_PIG	3.6±3.7	3.2±1.8	0.9	0.852	0.925
Cathepsin G	F1SGS1_PIG	0.7±0.9	0.0±0.0	0.0	n/a	n/a
Chitinase-3-like protein 1	CH3L1_PIG	0.0±0.0	0.0±0.0	n/a	n/a	n/a
Chondroitin sulfate proteoglycan 4*	CSPG4_HUMAN	0.1±0.2	0.0±0.0	0.0	n/a	n/a
Clusterin	CLUS_PIG	95.9±46.7	19.7±8.5	0.2	**0.044**	0.301
Collagen alpha-1 (I)	CO1A1_PIG	2055.2±391.6	2088.2±468.7	1.0	0.926	0.958
Collagen alpha-1 (II)*	I3LSV6_PIG	82.5±12.5	67.2±16.1	0.8	0.248	0.514
Collagen alpha-1 (III)	F1RYI8_PIG	252.0±85.1	431.9±112.0	1.7	0.087	0.384
Collagen alpha-1 (IV)	M3V819_PIG	4.3±2.7	5.2±1.7	1.2	0.628	0.778
Collagen alpha-1 (V)	F1S021_PIG	75.2±29.8	96.9±37.1	1.3	0.453	0.640
Collagen alpha-1 (VI)	CO6A1_PIG	6.2±4.7	7.9±6.9	1.3	0.746	0.851
Collagen alpha-1 (VIII)	F1SKX7_PIG	0.2±0.4	0.2±0.3	1.1	n/a	n/a
Collagen alpha-1 (XI)	F1S571_PIG	42.1±14.8	56.5±23.0	1.3	0.408	0.595
Collagen alpha-1 (XII)	COCA1_PIG	6.1±2.5	3.4±3.6	0.6	0.338	0.548
Collagen alpha-1 (XV)	COFA1_PIG	4.4±3.8	6.8±4.6	1.6	0.491	0.672
Collagen alpha-1 (XVIII)	COIA1_PIG	10.6±4.7	10.5±1.4	1.0	0.990	0.990
Collagen alpha-1 (XX)	COKA1_PIG	12.5±4.4	21.3±4.4	1.7	0.053	0.301
Collagen alpha-2 (I)	F1SFA7_PIG	1819.5±446.0	2184.2±434.5	1.2	0.332	0.548
Collagen alpha-2 (IV)	F1RLL9_PIG	5.4±6.5	5.6±2.6	1.0	0.961	0.972
Collagen alpha-2 (V)	Q59IP2_PIG	75.8±40.3	84.1±59.3	1.1	0.846	0.925
Collagen alpha-2 (VI)	I3LQ84_PIG	24.5±16.6	31.7±22.6	1.3	0.669	0.804
Collagen alpha-2 (XI)*	A5D9K7_PIG	24.6±9.4	25.0±7.5	1.0	0.951	0.972
Collagen alpha-3 (V)	Q59IP1_PIG	0.6±0.4	1.2±0.9	2.0	0.338	0.548
Collagen alpha-3 (VI)	I3LUR7_PIG	58.0±38.7	63.2±66.2	1.1	0.911	0.953
Collagen alpha-6 (VI)	CO6A6_PIG	0.2±0.3	0.0±0.0	0.0	n/a	n/a
Complement C3	CO3_PIG	12.3±8.9	1.8±2.1	0.1	0.097	0.384
Complement component C9	A0SEG9_PIG	0.0±0.0	0.0±0.0	n/a	n/a	n/a
Connective tissue growth factor	CTGF_PIG	0.2±0.2	0.0±0.0	0.0	n/a	n/a
Decorin	PGS2_PIG	369.9±68.6	569.0±248.3	1.5	0.298	0.548
Dermatopontin	DERM_PIG	36.2±8.6	74.7±13.9	2.1	**0.022**	0.288
Dystroglycan	I3LD20_PIG	0.5±0.3	2.2±0.6	4.5	**0.026**	0.288
EMILIN-1	F1SDQ5_PIG	9.4±4.2	14.5±3.6	1.5	0.148	0.420
Fibrillin-1	FBN1_PIG	0.0±0.0	0.0±0.0	n/a	n/a	n/a
Fibrinogen beta chain	F1RX37_PIG	1514.5±1633.6	17.6±25.7	0.0	0.164	0.429
Fibrinogen gamma chain	F1RX35_PIG	1214.6±1333.0	16.2±19.1	0.0	0.170	0.429

Fibromodulin	F1S6B5_PIG	76.2±40.4	273.4±67.2	3.6	**0.019**	0.288
Fibronectin	F1SS24_PIG	996.9±285.9	314.4±116.2	0.3	**0.011**	0.253
Fibronectin type III domain-containing protein 1	F1SB59_PIG	0.0±0.0	0.0±0.1	n/a	n/a	n/a
Fibulin-1	F1SM61_PIG	0.6±0.4	0.3±0.3	0.4	0.269	0.519
Fibulin-2	FBLN2_PIG	0.2±0.3	0.2±0.4	1.1	n/a	n/a
Fibulin-3	F8SIP2_PIG	0.0±0.0	0.0±0.0	n/a	n/a	n/a
Fibulin-5	F1SD87_PIG	10.0±5.2	14.0±8.2	1.4	0.511	0.689
Galectin-1	LEG1_PIG	22.8±13.4	44.5±6.0	2.0	**0.041**	0.301
Galectin-3	A3EX84_PIG	0.1±0.2	0.2±0.1	2.0	n/a	n/a
Galectin-3-binding protein	M3V7X9_PIG	4.7±7.0	0.0±0.0	0.0	n/a	n/a
Gelsolin	GELS_PIG	164.9±7.5	250.3±39.5	1.5	0.061	0.301
Hyaluronan and proteoglycan link protein 1	HPLN1_PIG	6.1±3.8	9.8±4.3	1.6	0.302	0.548
Hyaluronan and proteoglycan link protein 3*	HPLN3_HUMAN	1.6±0.7	0.8±1.1	0.5	0.367	0.565
Hyaluronan and proteoglycan link protein 4*	HPLN4_HUMAN	0.3±0.6	0.0±0.0	0.0	n/a	n/a
Insulin-like growth factor-binding protein 7	C7EDN1_PIG	2.7±1.7	6.4±5.0	2.4	0.329	0.548
Inter-alpha-trypsin inhibitor heavy chain H2	ITIH2_PIG	7.2±1.4	2.1±1.6	0.3	**0.011**	0.253
Lactadherin	MFGM_PIG	13.5±7.6	11.8±2.5	0.9	0.686	0.815
Laminin subunit alpha-4	F1RZM4_PIG	6.5±4.6	9.1±5.2	1.4	0.522	0.694
Laminin subunit beta-1	F1SAE9_PIG	3.6±2.6	7.6±1.7	2.1	0.060	0.301
Laminin subunit beta-2	F1SPT5_PIG	9.4±2.0	10.5±3.3	1.1	0.630	0.778
Laminin subunit gamma-1	F1S663_PIG	41.1±7.8	51.6±4.7	1.3	0.080	0.374
Latent-TGFß-binding protein 1	F1S405_PIG	0.5±0.5	0.2±0.3	0.3	0.272	0.519
Latent-TGFß-binding protein 2	F1S2T5_PIG	3.3±2.5	2.3±1.5	0.7	0.558	0.720
Latent-TGFß-binding protein 4	LTBP4_PIG	1.0±1.9	2.9±0.9	3.1	n/a	n/a
Leukocyte elastase inhibitor	ILEU_PIG	1.3±1.7	0.0±0.0	0.0	n/a	n/a
Lipoprotein lipase*	LIPL_PIG	1.4±1.0	0.0±0.0	0.0	n/a	n/a
Lumican	F1SQ09_PIG	201.5±83.1	274.7±84.1	1.4	0.310	0.548
Lysyl oxidase homolog 1	F1SIC9_PIG	2.3±2.5	8.9±5.1	3.9	0.140	0.417
Macrophage-capping protein	F1SVB0_PIG	0.0±0.0	0.1±0.1	n/a	n/a	n/a
Matrix Gla protein	MGP_PIG	1.1±0.6	1.3±0.7	1.2	0.726	0.840
Matrix-remodeling-associated protein5	F1RZ07_PIG	5.8±7.9	2.3±3.1	0.4	0.467	0.649
Mimecan	I3L9T6_PIG	222.1±65.9	359.4±47.8	1.6	**0.024**	0.288
Myeloperoxidase	K7GRV6_PIG	5.0±5.4	0.0±0.0	0.0	n/a	n/a
Nidogen-1	NID1_PIG	1.3±0.3	2.2±0.6	1.7	0.095	0.384
Nidogen-2	F1SFF3_PIG	107.0±40.5	191.0±42.9	1.8	0.055	0.301
Papilin	F1S3J7_PIG	1.5±2.1	0.6±0.5	0.4	0.428	0.614
Pentraxin-related protein PTX3*	F1SJM0_PIG	1.6±0.5	0.0±0.0	0.0	n/a	n/a
Peptidyl-prolyl cis-trans isomerase A	PPIA_PIG	3.5±2.1	3.2±0.8	0.9	0.780	0.879
Periostin	F1RS37_PIG	18.4±18.6	24.1±13.7	1.3	0.660	0.804
Perlecan	PGBM_PIG	223.6±75.7	314.7±59.1	1.4	0.134	0.413
Peroxidasin homolog	I3LDA4_PIG	0.3±0.3	0.1±0.2	0.4	n/a	n/a
Pigment epithelium-derived factor	Q0PM28_PIG	0.8±1.1	0.7±0.5	0.9	0.876	0.939
Plasminogen	PLMN_PIG	28.7±24.0	0.4±0.6	0.0	0.099	0.384
Podocan	I3LEB7_PIG	0.9±0.8	3.7±1.3	4.3	**0.045**	0.301
Procollagen C-endopeptidase enchancer 1	I3LEE6_PIG	0.4±0.5	0.9±0.7	2.3	0.373	0.565
Prolargin	F1S6B4_PIG	203.8±44.9	319.0±81.4	1.6	0.117	0.403
Prolow-density lipoprotein receptor-related protein 1	K9IVL7_PIG	3.7±1.9	1.8±1.4	0.5	0.189	0.431
Properdin	K7GQR1_PIG	0.7±0.6	0.1±0.1	0.2	0.152	0.420
Prophenin and tritrpticin precursor	PF11_PIG	116.0±100.2	0.3±0.5	0.0	n/a	n/a
Proteoglycan 4	I3L5Z3_PIG	0.0±0.0	0.0±0.0	n/a	n/a	n/a
RPE-spondin	RPESP_PIG	6.6±1.5	7.6±0.6	1.2	0.274	0.519

Secreted frizzled-related protein 1	I3LB66_PIG	0.0±0.0	0.8±0.4	n/a	n/a	n/a
Serine protease HTRA1	F1SEH4_PIG	5.9±3.4	24.5±17.8	4.2	0.208	0.463
Serotransferrin	TRFE_PIG	3.3±3.0	0.2±0.3	0.1	n/a	n/a
Serum amyloid P-component	SAMP_PIG	0.2±0.3	0.0±0.0	0.0	n/a	n/a
SPARC	SPRC_PIG	1.4±1.6	3.1±0.6	2.2	0.122	0.403
Spondin-1	SPON1_PIG	0.2±0.4	0.5±0.4	2.8	n/a	n/a
Sulfhydryl oxidase	F1S682_PIG	0.1±0.2	0.0±0.0	0.0	n/a	n/a
Superoxide dismutase [Cu-Zn]	Q007T6_PIG	0.9±1.0	3.2±1.9	3.4	0.156	0.420
Target of Nesh-SH3*	F1SL03_PIG	1.0±1.7	2.7±2.2	2.7	0.339	0.548
Tenascin	TENA_PIG	81.9±36.9	36.0±30.0	0.4	0.131	0.413
Tenascin XB*	A5A8W4_PIG	2.8±4.7	3.4±2.3	1.2	0.831	0.924
Tetranectin	F1SRC8_PIG	1.1±1.0	1.4±0.7	1.2	0.698	0.818
TGFß-1*	TGFB1_PIG	0.0±0.0	0.0±0.0	n/a	n/a	n/a
TGFß-3*	K7GSJ9_PIG	0.0±0.0	0.0±0.0	n/a	n/a	n/a
TGFß-induced protein ig-h3	BGH3_PIG	34.6±17.0	16.1±8.1	0.5	0.121	0.403
**Throbospondin-1**	K7GPJ3_PIG	5.7±0.9	0.4±0.4	0.1	**0.000**	**0.024**
Tryptase	TRYT_PIG	0.0±0.0	0.0±0.0	n/a	n/a	n/a
Tubulointerstitial nephritis antigen-like	F1SVA2_PIG	58.3±3.7	67.8±14.1	1.2	0.362	0.565
Versican	F1REZ2_PIG	106.1±58.1	142.6±42.5	1.3	0.381	0.566
Vitamin D-binding protein	I3LN42_PIG	7.3±7.7	0.5±0.5	0.1	0.173	0.429
Vitronectin	VTNC_PIG	149.0±66.3	43.5±29.8	0.3	**0.043**	0.301
von Willebrand factor A domain-containing protein 1	F1RJE3_PIG	11.1±2.7	16.8±5.1	1.5	0.183	0.431
WD repeat-containing protein 1	K9IVR7_PIG	0.0±0.1	0.1±0.0	1.4	0.598	0.760

P values for differential expression between BA early and BA late are based on unpaired Student's *t*-tests with unequal variance (early: n=4 [day 1 + 3], late: n=3 [day 14 + 28]). *T*-test was not performed if a protein was undetectable in the majority of samples from 1 of the 2 groups compared. Results in bold indicate *P* < 0.05. Values are average (Av) total ion current (TIC) x 10^6 ± standard deviation (SD). n/a denotes not applicable, FC denotes fold change. FDR denotes false discovery rate. The FDR threshold was set at 10%. Protein changes with *P* <0.05 and a FDR of <10% are highlighted in bold. Proteins only identified in the media, but not neointima are marked with*.

Supplemental Table V. Significant extracellular protein changes between BMS or DES day 28 vs. BA late.

Identified Proteins	UniProt ID	MW (kDa)	BMS/BA			DES/BA		
			FC	*P*	FDR	FC	*P*	FDR
RPE-spondin	RPESP_PIG	32	0.2	**0.006**	0.172	0.3	**0.001**	**0.012**
Laminin subunit gamma-1	F1S663_PIG	177	0.9	0.562	0.697	0.3	**0.001**	**0.012**
Galectin-3	A3EX84_PIG	27	23.4	0.395	0.612	6.8	**0.002**	**0.012**
Collagen alpha-1 (XI)	F1S571_PIG	159	1.8	0.272	0.584	3.4	**0.002**	**0.012**
Periostin	F1RS37_PIG	93	6.1	0.394	0.612	11.9	**0.002**	**0.012**
Biglycan	K7GP55_PIG	41	1.5	0.167	0.584	2.0	**0.004**	**0.021**
Collagen alpha-1 (V)	F1S021_PIG	182	1.8	0.267	0.584	2.6	**0.006**	**0.021**
Latent-TGFß-binding protein 2	F1S2T5_PIG	196	1.2	0.833	0.860	3.9	**0.006**	**0.021**
Collagen alpha-1 (XVIII)	COIA1_PIG	62	0.6	**0.027**	0.256	0.2	**0.007**	**0.021**
Collagen alpha-1 (I)	CO1A1_PIG	100	1.3	0.584	0.697	1.9	**0.007**	**0.021**
Apolipoprotein H	I3LGN5_PIG	29	1.5	0.463	0.648	3.1	**0.008**	**0.021**
Dermatopontin	DERM_PIG	22	1.7	0.328	0.599	2.0	**0.008**	**0.021**
Collagen alpha-2 (I)	F1SFA7_PIG	129	1.2	0.639	0.734	2.0	**0.010**	**0.024**
Laminin subunit beta-1	F1SAE9_PIG	199	1.2	0.197	0.584	0.3	**0.013**	**0.028**
Fibronectin	F1SS24_PIG	272	1.6	0.243	0.584	2.9	**0.014**	**0.028**
Aggrecan	F1SKR0_PIG	238	1.6	0.250	0.584	3.0	**0.017**	**0.032**
Collagen alpha-1 (II)	I3LSV6_PIG	130	1.0	0.961	0.961	1.7	**0.018**	**0.032**
Annexin A1	ANXA1_PIG	39	4.0	0.364	0.612	7.4	**0.022**	**0.038**
Collagen alpha-2 (XI)	A5D9K7_PIG	162	1.5	0.436	0.643	2.1	**0.024**	**0.040**
Collagen alpha-1 (XII)	COCA1_PIG	229	13.7	0.284	0.584	15.7	**0.026**	**0.041**
Collagen alpha-1 (VIII)	F1SKX7_PIG	73	2.6	0.301	0.584	15.3	**0.028**	**0.041**
Tetranectin	F1SRC8_PIG	22	1.7	0.118	0.523	4.9	**0.036**	**0.049**
Gelsolin	GELS_PIG	85	0.7	0.267	0.584	0.6	**0.036**	**0.049**
Agrin	I3LGD9_PIG	216	0.9	0.693	0.740	0.6	**0.040**	**0.050**
Nidogen-2	F1SFF3_PIG	152	0.9	0.687	0.740	0.5	**0.042**	**0.050**
Galectin-1	LEG1_PIG	15	0.7	0.080	0.411	0.6	**0.042**	**0.050**
Dystroglycan	I3LD20_PIG	95	0.3	**0.046**	0.285	0.4	**0.043**	**0.050**
Podocan	I3LEB7_PIG	72	0.8	0.502	0.648	0.3	**0.049**	**0.053**
Lactadherin	MFGM_PIG	46	1.6	0.498	0.648	0.5	**0.050**	**0.053**
Throbospondin-1	K7GPJ3_PIG	120	6.5	**0.031**	0.256	14.8	0.090	**0.093**
SPARC	SPRC_PIG	34	0.3	**0.033**	0.256	1.5	0.144	0.144

P value and fold change (FC) for changes between BMS or DES day 28 and BA late are shown. *P* values are based on unpaired Student's *t*-tests with unequal variance (n=3 [BMS day 28] or n=3 [DES day 28], n=3 [BA late]). *T*-test was not performed if a protein was undetectable in the majority of samples from 1 of the 2 groups compared. Results in bold indicate $P < 0.05$. FDR denotes false discovery rate. The FDR threshold was set at 10%.

Supplemental Table VI. Differentially expressed proteins between DES and BMS at different time points.

			Day 1			Day 3			Day 7			Day 28		
**Identified Proteins**	**UniProt ID**	**MW (kDa)**	**FC**	***P***	**FDR**	**FC**	***P***	**FDR**	**FC**	***P***	**FDR**	**FC**	***P***	**FDR**
Collagen alpha-2 (IV)	F1RLL9_PIG	161	0.6	0.362	0.683	0.6	0.338	0.676	1.2	0.707	0.868	0.3	**<0.001**	**0.008**
Laminin subunit beta-1	F1SAE9_PIG	199	0.6	0.432	0.683	1.0	0.975	0.975	0.7	0.639	0.868	0.2	**0.001**	**0.008**
Laminin subunit gamma-1	F1S663_PIG	177	0.9	0.741	0.803	1.1	0.440	0.770	1.1	0.753	0.868	0.3	**0.011**	**0.050**
SPARC	SPRC_PIG	34	1.3	n/a	n/a	0.1	**0.036**	0.201	0.9	0.802	0.868	5.6	**0.014**	**0.050**
Hyaluronan and proteoglycan link protein 1	HPLN1_PIG	40	1.7	0.427	0.683	2.8	**0.043**	0.201	1.5	0.419	0.868	0.3	**0.019**	**0.050**
Collagen alpha-1 (XVIII)	COIA1_PIG	62	0.8	0.540	0.683	1.0	0.921	0.975	1.1	0.788	0.868	0.2	**0.020**	**0.050**
Latent-TGFß-binding protein 2	F1S2T5_PIG	196	1.0	0.952	0.952	0.8	0.753	0.975	1.9	**0.003**	**0.045**	3.3	**0.026**	**0.054**
Collagen alpha-1 (VIII)	F1SKX7_PIG	73	0.6	n/a	n/a	2.0	n/a	n/a	1.2	0.645	0.868	5.9	**0.029**	**0.054**
Laminin subunit beta-2	F1SPT5_PIG	179	0.7	0.364	0.683	1.4	0.201	0.563	1.2	0.728	0.868	0.5	**0.038**	**0.057**
Tubulointerstitial nephritis antigen-like	F1SVA2_PIG	52	0.9	0.326	0.683	1.2	0.686	0.975	1.3	0.125	0.625	0.7	**0.038**	**0.057**
Aggrecan	F1SKR0_PIG	238	0.8	0.523	0.683	4.1	0.263	0.614	1.0	0.971	0.971	1.9	**0.044**	**0.060**
TGFß-induced protein ig-h3	BGH3_PIG	74	0.5	**0.045**	0.293	1.0	0.950	0.975	2.4	0.368	0.868	0.3	0.206	0.258
Prophenin and tritrpticin precursor	PF11_PIG	24	1.5	0.388	0.683	11.1	0.199	0.563	4.6	**0.010**	**0.075**	0.1	0.467	0.539
Collagen alpha-1 (XII)	COCA1_PIG	229	0.8	**0.045**	0.293	1.1	0.849	0.975	0.8	0.810	0.868	1.1	0.846	0.906
Vitronectin	VTNC_PIG	53	0.7	0.578	0.683	2.9	**0.019**	0.201	1.6	0.440	0.868	0.9	0.947	0.947

P values between DES and BMS at each time point are based on unpaired *t*-tests with unequal variance (n=3 [BMS day 1/3/7/28], n=3 [DES 1/3/7/28]). *T*-test was not performed if a protein was undetectable in the majority of samples from 1 of the 2 groups compared. Only proteins with at least one significant result at any time point are shown. Results in bold indicate $P < 0.05$. n/a denotes not applicable, FC denotes fold change (DES/BMS). FDR denotes false discovery rate. The FDR threshold was set at 10%.

Supplemental Table VII. Transitions for targeted proteomics in human vessels.

Protein	Proteotypic peptide + position	Average RT (min)	Precursor m/z	Precursor z	Fragment ion	Fragment ion m/z
**Aggrecan**	C[+57]GGNLLGVR	45.0	473.2504	2	y3	331.21
	C318 - R326				y4	444.29
					y5	557.38
					y6	671.42
					y7	728.44
					y8	785.46
**Versican**	LATVGELQAAWR	124.6	657.8619	2	y4	503.27
	L277 - R288				y5	631.33
					y6	744.42
					y7	873.46
					y8	930.48
					y9	1029.55
					y10	1130.60
					y11	1201.63
**Decorin**	NLHALILVNNK	67.6	416.9189	3	y3	375.20
	N106 - K116				y4	474.27
					y5	587.35
					y6	700.44

Supplemental Table VIII. Aortic extracellular proteins identified by proteomics analysis in mice lacking the catalytic domain of *Adamts5* (*Adamts5* Δcat) compared to controls.

Identified Proteins	UniProt ID	MW (kDa)	WT	*Adamts5* Δcat	*P*	FC (Δcat /WT)
			Total ion current x 10^6 Av±SD			
Adipocyte enhancer-binding protein 1	AEBP1_MOUSE	128	64.1±31.6	58.4±25.3	0.746	0.9
Adiponectin	ADIPO_MOUSE	27	40.9±9.0	33.2±20.2	0.465	0.8
**Aggrecan**	PGCA_MOUSE	222	176.9±30.6	255.3±56.3	**0.032**	1.4
Agrin	AGRIN_MOUSE	208	34.0±16.3	33.0±13.1	0.914	1.0
Alpha-2-HS-glycoprotein	FETUA_MOUSE	37	11.8±14.4	15.4±14.3	0.689	1.3
Amyloid beta A4 protein	A4_MOUSE	87	18.1±15.0	11.1±10.9	0.397	0.6
Annexin A2	ANXA2_MOUSE	39	41.3±26.3	58.4±46.5	0.492	1.4
Apolipoprotein A-I	APOA1_MOUSE	31	43.3±21.6	53.3±32.3	0.573	1.2
Apolipoprotein A-IV	APOA4_MOUSE	45	21.6±13.2	26.7±7.4	0.450	1.2
Apolipoprotein E	APOE_MOUSE	36	0.0±0.0	4.7±10.6	n/a	n/a
Asporin	ASPN_MOUSE	43	235.9±42.9	236.6±39.4	0.976	1.0
Basal cell adhesion molecule	BCAM_MOUSE	68	180.7±43.3	185.7±33.6	0.835	1.0
Basement membrane-specific heparan sulfate proteoglycan core protein	PGBM_MOUSE	398	2277.5±241.7	2497.0±107.5	0.085	1.1
Beta-2-glycoprotein 1	APOH_MOUSE	39	54.3±19.6	64.0±16.8	0.400	1.2
Biglycan	PGS1_MOUSE	42	2026.4±473.1	1924.8±417.8	0.714	0.9
Cadherin-13	CAD13_MOUSE	78	24.6±12.0	30.7±10.3	0.388	1.2
Cathepsin D	CATD_MOUSE	45	21.1±5.4	15.8±11.7	0.394	0.7
Cell surface glycoprotein MUC18	MUC18_MOUSE	72	16.7±14.0	19.7±12.4	0.719	1.2
Chondroitin sulfate proteoglycan 4	CSPG4_MOUSE	252	14.8±12.7	6.1±8.3	n/a	0.4
Chymase	CMA1_MOUSE	28	76.0±27.3	79.1±27.5	0.854	1.0
Clusterin	CLUS_MOUSE	52	80.6±14.8	93.2±31.9	0.450	1.2
Collagen alpha-1 (I)	CO1A1_MOUSE	138	17254.0±2798.8	21811.8±4876.9	0.113	1.3
Collagen alpha-1 (II)	CO2A1_MOUSE	142	645.2±292.1	562.5±183.9	0.583	0.9
Collagen alpha-1 (III)	CO3A1_MOUSE	139	2265.9±1681.1	1177.9±635.7	0.189	0.5
Collagen alpha-1 (IV)	CO4A1_MOUSE	161	91.4±30.3	61.6±16.9	0.074	0.7
Collagen alpha-1 (V)	CO5A1_MOUSE	184	281.7±52.1	290.4±66.5	0.817	1.0
Collagen alpha-1 (VI)	CO6A1_MOUSE	108	213.0±64.7	209.5±106.6	0.951	1.0
Collagen alpha-1 (XI)	COBA1_MOUSE	181	62.6±54.8	0.0±0.0	n/a	0.0
Collagen alpha-1 (XIV)	COEA1_MOUSE	193	14.5±17.3	5.7±12.8	n/a	0.4
Collagen alpha-1 (XV)	COFA1_MOUSE	140	137.4±35.4	151.9±42.4	0.560	1.1
Collagen alpha-1 (XVIII)	COIA1_MOUSE	182	295.6±66.3	277.6±64.0	0.660	0.9
Collagen alpha-2 (I)	CO1A2_MOUSE	130	11217.6±3451.6	11447.8±2260.1	0.897	1.0
Collagen alpha-2 (IV)	CO4A2_MOUSE	167	36.9±26.3	53.8±31.3	0.367	1.5
Collagen alpha-2 (V)	CO5A2_MOUSE	145	408.7±94.8	442.8±135.1	0.649	1.1
Collagen alpha-2 (VI)	CO6A2_MOUSE	110	122.8±66.3	81.2±54.4	0.282	0.7
Collagen alpha-2 (XI)	COBA2_MOUSE	172	7.5±18.3	0.0±0.0	n/a	0.0
Collagen alpha-5 (VI)	CO6A5_MOUSE	290	5.7±13.8	0.0±0.0	n/a	0.0
Collagen alpha-6 (VI)	CO6A6_MOUSE	246	81.1±50.5	91.9±86.1	0.812	1.1
Connective tissue growth factor	CTGF_MOUSE	38	2.6±6.4	11.2±15.7	n/a	4.3
Decorin	PGS2_MOUSE	40	1366.3±105.8	1401.5±270.2	0.795	1.0
Dermatopontin	DERM_MOUSE	24	151.7±30.2	136.3±39.5	0.495	0.9
Destrin	DEST_MOUSE	19	277.8±70.8	326.5±62.5	0.257	1.2
Dystroglycan	DAG1_MOUSE	97	31.3±15.6	57.0±30.4	0.140	1.8
Elastin	ELN_MOUSE	72	44.4±19.8	44.0±19.3	0.978	1.0
EMILIN-1	EMIL1_MOUSE	108	4.2±10.3	0.0±0.0	n/a	0.0
Extracellular superoxide dismutase [Cu-Zn]	SODE_MOUSE	27	430.7±126.7	416.8±106.0	0.847	1.0
Fibrillin-1	FBN1_MOUSE	312	25.2±25.4	0.0±0.0	n/a	0.0
Fibrinogen alpha chain	FIBA_MOUSE	87	38.7±26.0	23.2±27.0	0.360	0.6

Fibrinogen beta chain	FIBB_MOUSE	55	33.8±29.5	30.0±41.8	0.868	0.9
Fibrinogen gamma chain	FIBG_MOUSE	49	60.0±56.4	40.0±34.1	0.487	0.7
Fibromodulin	FMOD_MOUSE	43	9.6±7.6	6.9±7.4	0.578	0.7
Fibronectin	FINC_MOUSE	273	1061.0±222.9	951.9±142.2	0.353	0.9
Fibulin-5	FBLN5_MOUSE	50	62.2±27.6	73.3±23.2	0.487	1.2
Galectin-1	LEG1_MOUSE	15	224.4±39.5	265.8±56.3	0.209	1.2
Galectin-3	LEG3_MOUSE	28	14.3±7.3	30.5±29.9	0.297	2.1
Hyaluronan and proteoglycan link protein 1	HPLN1_MOUSE	40	8.5±8.1	11.2±15.7	n/a	1.3
Insulin-like growth factor-binding protein 7	IBP7_MOUSE	29	154.1±49.6	129.6±35.5	0.367	0.8
Inter-alpha-trypsin inhibitor heavy chain H1	ITIH1_MOUSE	101	5.7±11.2	4.6±6.4	n/a	0.8
Lactadherin	MFGM_MOUSE	51	384.5±33.4	387.7±63.6	0.923	1.0
Laminin subunit alpha-2	LAMA2_MOUSE	344	1.8±4.4	0.0±0.0	n/a	0.0
Laminin subunit alpha-4	LAMA4_MOUSE	202	43.6±8.6	37.0±25.6	0.611	0.9
Laminin subunit alpha-5	LAMA5_MOUSE	404	48.3±13.5	35.5±20.4	0.272	0.7
Laminin subunit beta-2	LAMB2_MOUSE	197	113.1±57.2	96.3±58.5	0.644	0.9
Laminin subunit gamma-1	LAMC1_MOUSE	177	113.4±28.6	91.6±15.7	0.148	0.8
Latent-TGFß-binding protein 1	LTBP1_MOUSE	187	47.3±15.7	50.1±13.6	0.756	1.1
Latent-TGFß-binding protein 4	LTBP4_MOUSE	179	456.7±66.7	429.5±95.6	0.608	0.9
Lumican	LUM_MOUSE	38	1799.7±751.8	1778.6±220.8	0.950	1.0
Lysyl oxidase homolog 1	LOXL1_MOUSE	67	368.2±63.6	376.3±39.2	0.802	1.0
Matrin-3	MATR3_MOUSE	95	1.4±3.4	0.0±0.0	n/a	0.0
Microfibril-associated glycoprotein 4	MFAP4_MOUSE	29	101.6±32.5	127.7±31.3	0.210	1.3
Microfibrillar-associated protein 5	MFAP5_MOUSE	19	66.0±70.8	88.8±90.0	0.658	1.3
**Mimecan**	MIME_MOUSE	34	1299.0±180.9	1605.9±194.2	**0.026**	1.2
Nidogen-1	NID1_MOUSE	137	193.9±65.0	192.4±24.6	0.959	1.0
Nidogen-2	NID2_MOUSE	154	123.2±35.4	109.1±36.4	0.533	0.9
Periostin	POSTN_MOUSE	93	182.1±59.7	193.5±156.6	0.884	1.1
Prolargin	PRELP_MOUSE	43	578.3±87.4	622.8±159.5	0.597	1.1
Prosaposin	SAP_MOUSE	61	31.9±24.8	15.2±21.6	n/a	0.5
Protein S100-A10	S10AA_MOUSE	11	15.8±24.4	0.0±0.0	n/a	0.0
Protein S100-A11	S10AB_MOUSE	11	0.0±0.0	7.4±10.1	n/a	n/a
Secreted frizzled-related protein 1	SFRP1_MOUSE	35	1.0±2.5	0.0±0.0	n/a	0.0
Serine protease HTRA1	HTRA1_MOUSE	51	169.9±40.2	197.7±38.0	0.270	1.2
SPARC	SPRC_MOUSE	34	2.9±4.6	3.2±7.2	n/a	1.1
Tenascin	TENA_MOUSE	232	6.2±9.7	1.8±4.0	n/a	0.3
Tetranectin	TETN_MOUSE	22	0.0±0.0	2.4±5.3	n/a	n/a
TGFß-induced protein ig-h3	BGH3_MOUSE	75	24.2±16.2	9.8±17.5	n/a	0.4
Thrombospondin type-1 domain-containing protein 4	THSD4_MOUSE	113	44.1±26.6	64.0±21.0	0.198	1.5
Versican	CSPG2_MOUSE	367	254.8±25.0	240.0±36.3	0.467	0.9
Vitamin D-binding protein	VTDB_MOUSE	54	0.0±0.0	3.5±7.8	n/a	n/a
**Vitronectin**	VTNC_MOUSE	55	71.5±22.0	115.2±31.8	**0.036**	1.6

P values for differential expression between aortas of wild type controls (WT) and mice lacking the catalytic domain of *Adamts5* (*Adamts5* Δcat) are based on unpaired Student's *t*-tests with unequal variance (n=6 [WT] and n=5 [*Adamts5* Δcat]). *T*-test was not performed if a protein was undetectable in the majority of samples from 1 of the 2 groups compared. Results in bold indicate $P < 0.05$. Values are average (Av) total ion current (TIC) x 10^6 ± standard deviation (SD). n/a denotes not applicable; FC denotes fold change.

Supplemental Table IX. Transitions for targeted proteomics in murine vessels.

Protein	Proteotypic peptide	Amino acid position	Collision energy	Retention time (min)	m/z light	m/z heavy	charge state
**Aggrecan**	GDPETSVSGVGDDFSGLPSGK	G1172 – K1192	37.1	n/a	1004.4607	1007.4708	2
	TVYLYPN[+3]QTGLPDPLSK	T661 – K677	35.3	163.22	954.9956	958.0057	2
**Versican**	VSVPTHPDDVGDASLTMVK	V101 – K119	36.4	92.82	984.4908	987.5008	2
	VSVPTHPDDVGDASLTMVK	V101 – K119	27.8	92.82	656.6629	658.6697	3
**Decorin**	DLHTLILVNNK	D101 – K111	24	84.52	640.3721	643.3821	2
	DLHTLILVNNK	D101 – K111	19.3	84.52	427.2505	429.2572	3
	NSGIENGAFQGLK	N183 – K195	25	n/a	667.8386	670.8487	2
	VVQC[+57]SDLGLDK	V59 – K69	23.2	37.16	617.3108	620.3209	2

SUPPLEMENTAL FIGURES

Supplemental Figure I. Overview of proteomics comparisons. The following proteomics comparisons were conducted in porcine tissue: a) Stent dependent changes in the neointima. b) Changes in BMS/DES/BA at different time points. c) Comparison stent vs. BA at late stage. d) BMS/DES dependent changes at each time point.

Supplemental Figure II. Reproducibility of LC-MS/MS. The neointima samples were run in duplicates by LC-MS/MS. The total spectral counts (R=0.998) and the sequence coverage (R=0.969) for each ECM protein correlated well between technical replicates. n=140 proteins x 14 samples (Pearson correlation). The Venn diagram displays the unique peptides identified either in both or only in the 1st and only in the 2nd analysis.

Supplemental Figure III. Database search. The publically available porcine databases do not cover the entire sequence of each protein. In addition, not all proteins are well annotated in porcine databases. Thus, a custom-made database containing porcine sequences of previously published ECM proteins was generated. This figure shows the identification of total spectral counts for selected proteins using different databases: The application of a hybrid database, which is the combination of a pig and human database, is superior to a pig or human database alone for identifying and quantifying proteins analysed by LC-MS/MS due to a higher sequence match. For many ECM proteins, such as fibronectin and decorin, which are well annotated and fully sequenced, the custom-made database does not offer a major improvement. However, for other proteins, such as collagen alpha-1 (I) and perlecan, which are not well annotated or fully sequenced, the custom-made database has a remarkable impact on the sequence coverage and protein quantification.

Supplemental Figure IV. Total identified proteins. Overall in the media and neointima a total of 151 unique ECM proteins were identified with a minimum of 2 high-confidence peptides. The Venn diagram depicts the findings in the media and neointima (114 common proteins, 11 only in the media, 26 only in the neointima).

Supplemental Figure V. **Densitometry DES at different time points**. Aggrecan NITEGE and versican DPEAAE neoepitopes generated by aggrecanase activity in DES at different time points. n=3 per time point. *P<0.05, **P<0.01, ***P<0.001 (1-way ANOVA [P<0.001] with Dunnett post hoc test to day 1).

Supplemental Figure VI. **Densitometry BMS vs DES day 28**. Differences between BMS and DES at day 28. n=4 per group. **P<0.01 (*t*-test with unequal variance).

Supplemental Figure VII. Aggrecan in human stented coronary artery. Co-localization of aggrecan (Alexa 633, displayed in green) and aggrecan NITEGE neoepitope (Alexa 568, displayed in red) in human stented coronary artery visualized by immunofluorescence. Overview image 20x, scale bar=500 μm; Zoomed-in areas 60x, scale bar=25 μm.

Supplemental Figure VIII. **Densitometry aggrecan in human vasculature.** Protein abundance quantified by densitometry of immunoblots for the aggrecan NITEGE neoepitope, versican and decorin in human thoracic aortas and saphenous veins. n=4 per group. *P<0.05 (*t* test with unequal variance).

Supplemental Figure IX. **Blood pressure measurements in mice**. Systolic and diastolic blood pressure measurements. n=7 (WT), n=5 (*Adamts5* Δcat), *t*-test with unequal variance.

Supplemental Figure X. **Cardiac output measurements in mice**. Left ventricular ejection fraction (LVEF) as measured by CMR. n=5 (WT), n=7 (*Adamts5* Δcat), *t*-test with unequal variance.

Supplemental Figure I

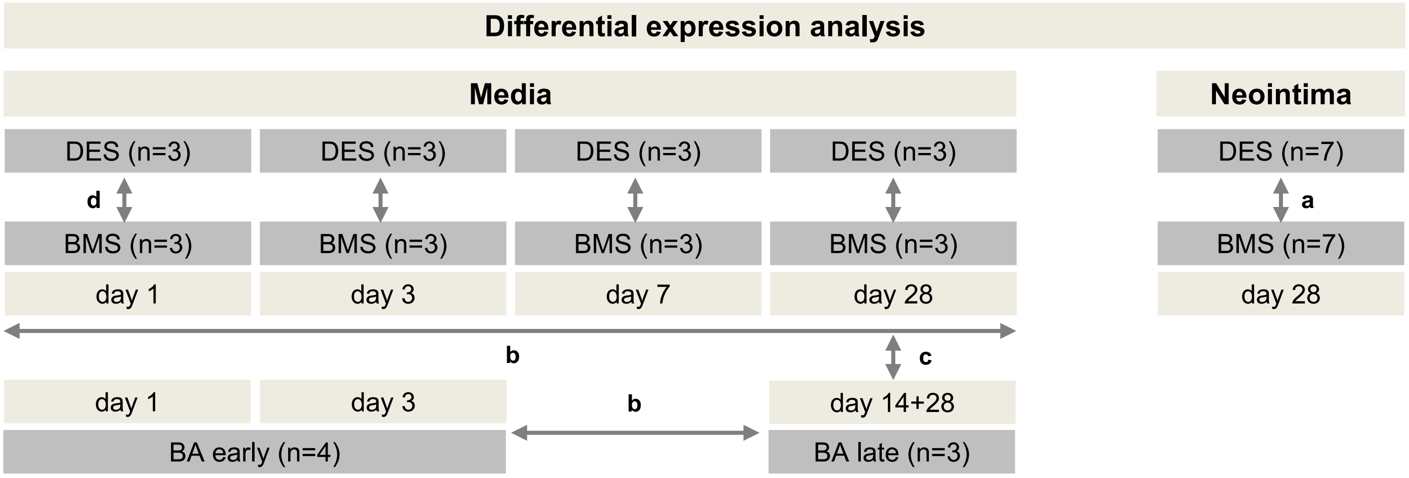

Supplemental Figure II

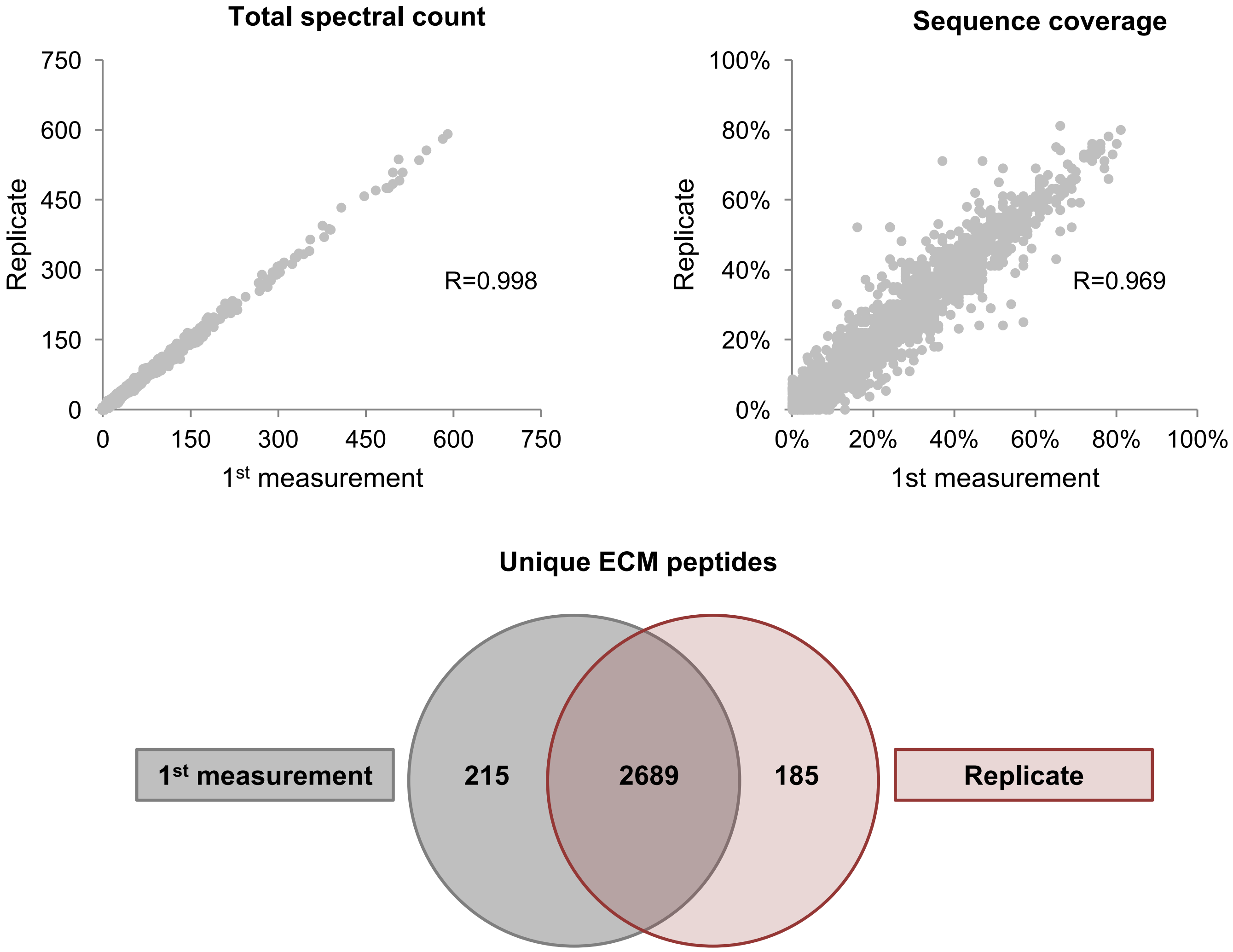

Supplemental Figure III

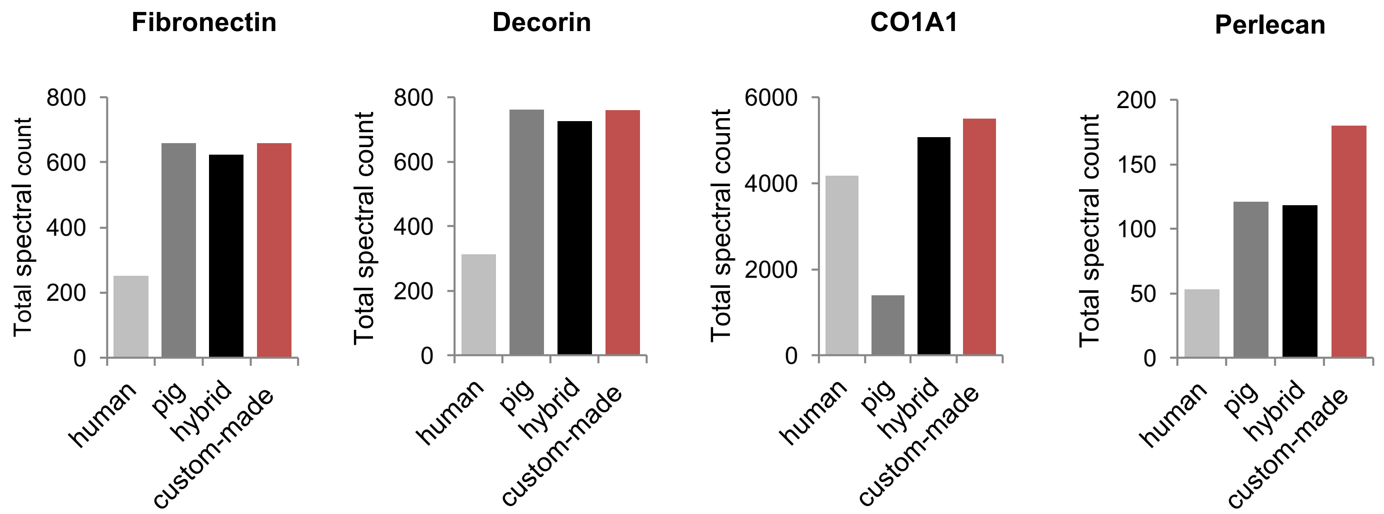

Supplemental Figure IV

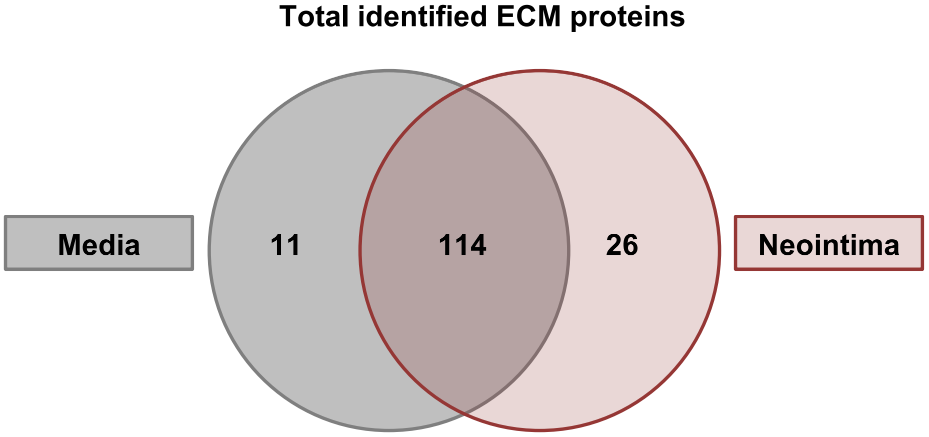

Supplemental Figure V

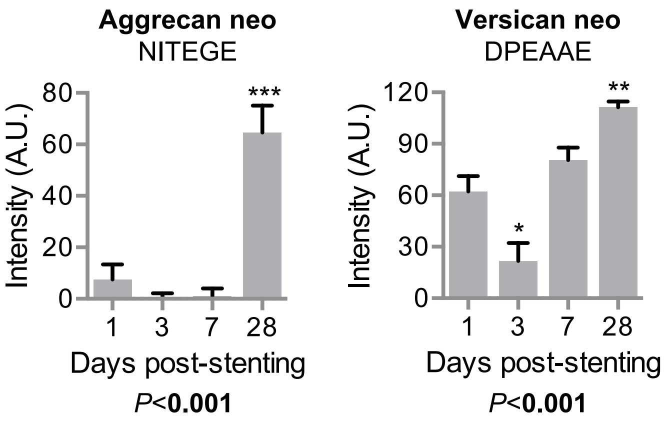

Supplemental Figure VI

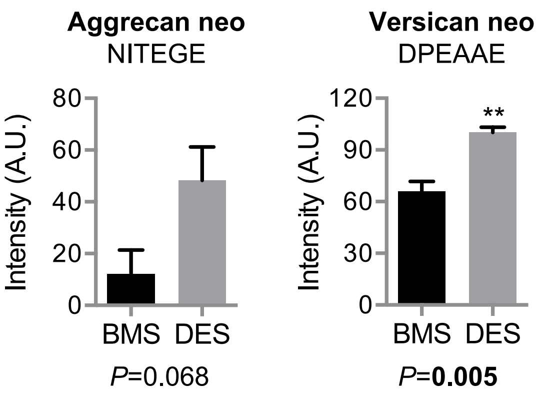

Supplemental Figure VII

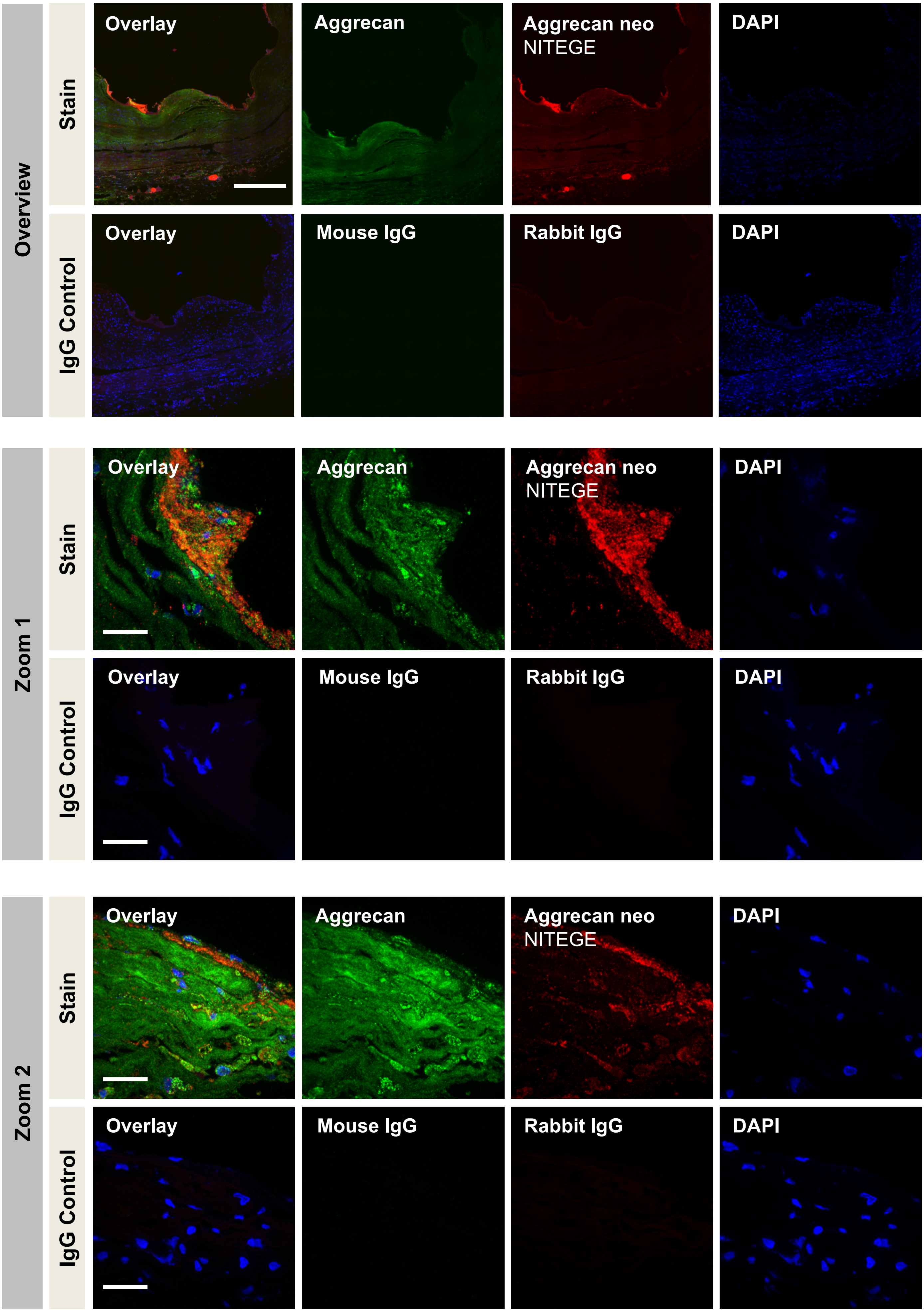

Supplemental Figure VIII

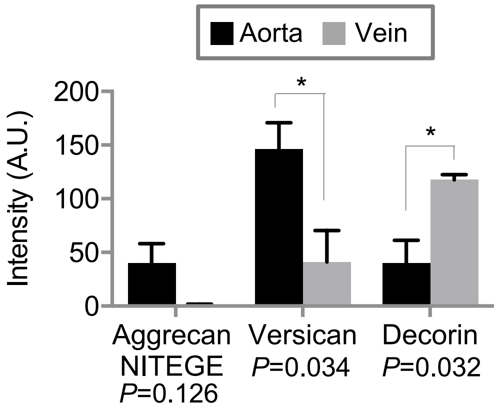

Supplemental Figure IX

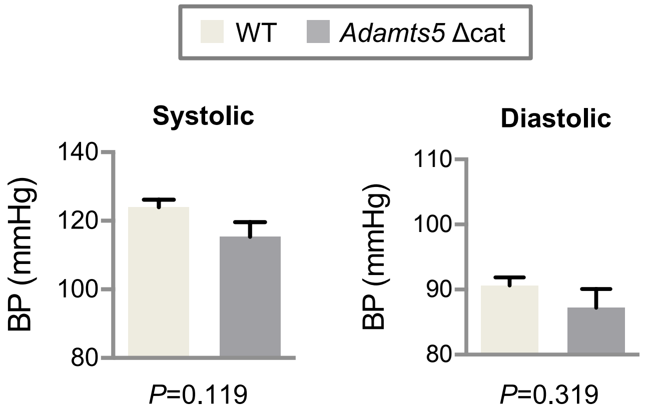

Supplemental Figure X

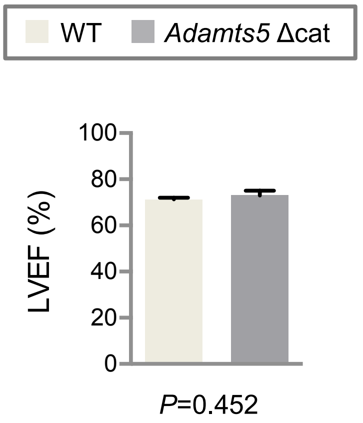

SUPPLEMENTAL REFERENCES

1. Gonzalo N, Serruys PW, Okamura T, van Beusekom HM, Garcia-Garcia HM, van Soest G, van der Giessen W, Regar E. Optical coherence tomography patterns of stent restenosis. *Am Heart J*. 2009;158:284-93. doi:10.1016/j.ahj.2009.06.004.

2. Barallobre-Barreiro J, Didangelos A, Schoendube FA, Drozdov I, Yin X, Fernandez-Caggiano M, Willeit P, Puntmann VO, Aldama-Lopez G, Shah AM, Domenech N, Mayr M. Proteomics analysis of cardiac extracellular matrix remodeling in a porcine model of ischemia/reperfusion injury. *Circulation*. 2012;125:789-802. doi:10.1161/CIRCULATIONAHA.111.056952.

3. Cuello F, Shankar-Hari M, Mayr U, Yin X, Marshall M, Suna G, Willeit P, Langley SR, Jayawardhana T, Zeller T, Terblanche M, Shah AM, Mayr M. Redox state of pentraxin 3 as a novel biomarker for resolution of inflammation and survival in sepsis. *Mol Cell Proteomics*. 2014;13:2545-57. doi:10.1074/mcp.M114.039446.

4. Didangelos A, Yin X, Mandal K, Baumert M, Jahangiri M, Mayr M. Proteomics characterization of extracellular space components in the human aorta. *Mol Cell Proteomics*. 2010;9:2048-62. doi:10.1074/mcp.M110.001693.

5. Kapustin AN, Chatrou ML, Drozdov I, Zheng Y, Davidson SM, Soong D, Furmanik M, Sanchis P, De Rosales RT, Alvarez-Hernandez D, Shroff R, Yin X, Muller K, Skepper JN, Mayr M, Reutelingsperger CP, Chester A, Bertazzo S, Schurgers LJ, Shanahan CM. Vascular smooth muscle cell calcification is mediated by regulated exosome secretion. *Circ Res*. 2015;116:1312-23. doi:10.1161/CIRCRESAHA.

6. Livak KJ and Schmittgen TD. Analysis of relative gene expression data using real-time quantitative PCR and the 2(-Delta Delta C(T)) Method. *Methods*. 2001;25:402-8. doi:10.1006/meth.2001.1262.

7. Zou Y, Dietrich H, Hu Y, Metzler B, Wick G, Xu Q. Mouse model of venous bypass graft arteriosclerosis. *Am J Pathol*. 1998;153:1301-10. doi: 10.1016/S0002-9440(10)65675-1.

8. Stanton H, Rogerson FM, East CJ, Golub SB, Lawlor KE, Meeker CT, Little CB, Last K, Farmer PJ, Campbell IK, Fourie AM, Fosang AJ. ADAMTS5 is the major aggrecanase in mouse cartilage in vivo and in vitro. *Nature*. 2005; 434:648-652. doi:10.1038/nature03417.
9. Schneider JE, Wiesmann F, Lygate CA, Neubauer S. How to perform an accurate assessment of cardiac function in mice using high-resolution magnetic resonance imaging. *J Cardiovasc Magn Reson*. 2006; 8:893-701. doi: 10.1080/10976640600723664.
